# Ionogels Derived from Fluorinated Ionic Liquids to Enhance Aqueous Drug Solubility for Local Drug Administration

**DOI:** 10.3390/gels8090594

**Published:** 2022-09-16

**Authors:** Carolina Hermida-Merino, David Cabaleiro, Carlos Gracia-Fernández, Jesus Valcarcel, José Antonio Vázquez, Noelia Sanz, Martín Pérez-Rodríguez, Maria Arenas-Moreira, Dipanjan Banerjee, Alessandro Longo, Carmen Moya-Lopez, Luis Lugo, Patrice Bourson, Ana B. Pereiro, Georges Salloum-Abou-Jaoude, Iván Bravo, Manuel M. Piñeiro, Daniel Hermida-Merino

**Affiliations:** 1 CINBIO, Departamento de Física Aplicada, Universidade de Vigo, Campus Lagoas-Marcosende, 36310 Vigo, Spain; 2 TA Instruments Waters Chromatography, Tres Cantos, 28760 Madrid, Spain; 3 Grupo de Reciclado y Valorización de Materiales Residuales (REVAL), Instituto de Investigaciones Marinas (IIM-CSIC), Eduardo Cabello 6, 36208 Vigo, Spain; 4 Grupo de Bioquímica de Alimentos, Instituto de Investigaciones Marinas (IIM-CSIC), Eduardo Cabello 6, 36208 Vigo, Spain; 5 Departamento de Química Física, Facultad de Farmacia, UCLM, 02071 Albacete, Spain; 6 DUBBLE CRG/ESRF, CEDEX, 38043 Grenoble, France; 7 Department of Chemistry, KU Leuven, Celestijnenlaan 200F, P.O. Box 2404, B-3001 Leuven, Belgium; 8 ID20, ESRF, 71 Avenue des Martyrs, 38000 Grenoble, France; 9 Istituto per lo Studio dei Materiali Nanostrutturati (ISMN)-CNR, UOS Palermo, Via Ugo La Malfa, 153, 90146 Palermo, Italy; 10 LMOPS, Centrale Supélec, Université de Lorraine, 57000 Metz, France; 11 LAQV, REQUIMTE, Departamento de Química, Faculdade de Ciências e Tecnologia, Universidade Nova de Lisboa, 2829-516 Caparica, Portugal; 12 Constellium C-TEC Technology Center, Parc Economique Centr’alp, 725 rue Aristide Bergès, CS10027, 38341 Voreppe, France

**Keywords:** gelatin, 1-ethyl-3-methylpyridinium perfluorobutanesulfonate, drug delivery, emulsions

## Abstract

Gelatin is a popular biopolymer for biomedical applications due to its harmless impact with a negligible inflammatory response in the host organism. Gelatin interacts with soluble molecules in aqueous media as ionic counterparts such as ionic liquids (ILs) to be used as cosolvents to generate the so-called Ionogels. The perfluorinated IL (FIL), 1-ethyl-3-methylpyridinium perfluorobutanesulfonate, has been selected as co-hydrosolvent for fish gelatin due to its low cytotoxicity and hydrophobicity aprotic polar structure to improve the drug aqueous solubility. A series of FIL/water emulsions with different FIL content and their corresponding shark gelatin/FIL Ionogel has been designed to enhance the drug solubility whilst retaining the mechanical structure and their nanostructure was probed by simultaneous SAXS/WAXS, FTIR and Raman spectroscopy, DSC and rheological experiments. Likewise, the FIL assisted the solubility of the antitumoural Doxorubicin whilst retaining the performing mechanical properties of the drug delivery system network for the drug storage as well as the local administration by a syringe. In addition, the different controlled release mechanisms of two different antitumoral such as Doxorubicin and Mithramycin from two different Ionogels formulations were compared to previous gelatin hydrogels which proved the key structure correlation required to attain specific therapeutic dosages.

## 1. Introduction

Significant efforts are continuously dedicated to generating biocompatible supports to design pharmaceutic formulations that enhance the poor solubility of hydrophobic drugs [1,2,3] whilst retaining the stability of the therapeutic agent over long storage periods. Besides, the control of the drug concentration in the bloodstream is a key parameter to designing an ideal nanocarrier as should maintain its effective therapeutic effect range and under the harmful threshold concentration in physiological conditions [4,5]. However, the characteristic biological half-life time of every drug urges the customization of the drug delivery system (DDS) [6,7] to deliver an effective concentration for the specific disease in a prolonged and controlled way in contrast with conventional dosage forms. In contrast, the recurrent drug intake of traditional treatments is required to assure its stability, activity and bioavailability.

The drug incorporation into biocompatible matrices [8] formed by biopolymers has frequently been adopted as a potential drug carrier for local administration due to their chemical architecture diversity and featured multifunctionality [9,10,11]. 

Particularly, responsive physical networks such as smart hydrogels combine the tunable mechanical properties for both drug storage and administration whilst offering bio scaffolding to promote complementary therapeutic cellular growth. The reversibility of the physical networks upon external fields such as shear or thermal stimuli, together with their relaxation and diffusion dynamics allows the hydrogels to feature ambivalent properties to both load and release drugs efficiently. However, the low solubility of hydrophobic drugs in aqueous media hampers their uptake to attain therapeutic dosage. Likewise, hydrogels based on gelatin were largely employed to generate therapeutic agents nanocarriers as a result of availability [12,13] in industrial waste as well as physicochemical properties and bioassimilation ability that is crucial for a wide range of medical applications [13,14,15,16,17,18]. Indeed, gelatin recovered from fishing by-products complies with the recommendations of the European Union to develop Circular Economy processes [19] as well as societal and religious concerns related to mammalian gelatin sources. In addition, fish gelatin physicochemical properties can be adjusted to overcome its inferior mechanical properties in comparison with mammalian counterparts, but also to maximize drug uptake and release efficiency for different therapeutic agents.

Gelatin hydrogels have been widely used for encapsulating numerous bioactive molecules, featuring higher intracellular uptake, as well as being suited for intravenous and local application in different areas of the body anatomy. The gelatin molecular architecture with both hydrophobic and hydrophilic groups enables the uptake and transport of a wide variety of therapeutic agents [20,21,22] whilst protecting them from degradation during the targeted delivery to minimize the harmful impact on healthy cells and other undesirable reactions [23]. Likewise, the tailoring of the drug carrier to therapeutic agent features determines if the dissolution, entrapping, encapsulation or attachment to the hydrogel dispersed network [22,23,24,25]. The hydrogels are generally polymeric mesh largely expanded, forming colloids in which the aqueous solution is dispersed [26]. The hydrophilic groups located either in the main chain or as pendant groups of the polymer backbone facilitate water retention whilst the physical or chemical cross-links between network chains impede their dissolution. Besides, bionanocomposites formed by co-hydrogelators are typically used to modify the network properties by generating double networks. Likewise, the strong interaction of gelatin with other water-soluble molecules such as ionic polymers, metal ion or ionic polymers drives them to afford the so-called ionic hydrogels [27,28,29,30,31].

Similarly, the use of co-solvents for tailoring the hydrogel networks such as alcohols has been previously explored to assist in the hydrogelator solubility of, for instance, polypeptides. Besides, ionic liquids (IL) were aroused as potential solvents for several physical and chemical processes as a result of their characteristic physicochemical features such as density, high thermal stability, low vapour pressure and the solvating ability for both polar and apolar chemicals. The general amphiphilic nature of the IL together with its non-ionizing character explain their solvation capability. In addition, ILs are considered green solvents [32] due to their biodegradability and low toxicity for sustainable chemical processes. 

However, the ILs solubility capacity depends to a great extent on the ion pair chemical composition, in particular, the substitution of the aliphatic chain for a fluorinated counterpart yields a multi nanosegregated structure related to the polar segment and both hydrogenated and fluorinated apolar fragment. Likewise, the electronic withdrawing nature of the fluorinated part and its interactions with the cation determine the solvation capability of the so-called fluorinated ILs (FILs). Remarkably, the solving capability of the ILs has been proved to improve the extraction and processing of biopolymers as well as their use as co-solvents to generate Ionogels [33,34,35]. Likewise, the biocompatibility of the FILs together with the solving ability of hydrophobic compounds allow them to stabilize different therapeutic agents and vaccines [36] that qualify them to be formulated for drug delivery systems or biomedical applications due to their antimicrobial activity that was found to surpass conventional antimicrobial agents [37].

The design of responsive multifunctional drug delivery hydrogel systems relies on the balance among the inter-intramolecular forces and the phase behavior between the dispersed phase and the continuous phase. Recent of our previous works on gelatin hydrogels derived from waste fisheries have proved [11,38,39,40] to feature promising mechanical properties for drug storage and local administration, although higher drug uptake should be favoured to prolongate the therapeutic dosage and thus, minimise the adverse frequent administration. Likewise, the physical properties of the hydrogel network modification are aimed in the current work to enhance drug solubility while retaining the biocompatibility of the system as well as the desired responsive mechanical properties suited for both storage and drug administration. Therefore, our approach is the addition of a co-solvent to the continuous aqueous phase that enhances the drug solubility that generally leads to heterogenous systems with emulsion structures. Ideally, the design proviso is to generate a liquid–liquid heterogenous system that should modify the physicochemical properties of the continuous phase without disrupting the subsequent hydrogel network upon the gelatin addition which is responsible for the storage and administration properties. Likewise, the incorporation of a dissolving agent for hydrophobic compounds in an aqueous solution yields heterogenous systems reminiscent of the amphiphilic surfactant activity [41] in a solution that commonly forms colloidal aggregates. Similarly, the nanostructure understanding of blends composed of water or alcohols with ionic liquids [42,43,44] as well as their phase behaviour was previously attended to be universalized, although with controversial results due to molecular structure dependence [45].In addition, ILs ionicity has been proven to assist in the extraction of biopolymers [46] and in particular gelatin [47]. Likewise, the use of novel, biocompatible and more environmentally friendly FILs possess positive prospects for turning proteins bioavailable and stable in physiological conditions. Particularly, the efficiency enhancement of drug proteins is of great interest to the pharmaceutic field due to their biocompatibility which facilitates their clinic approval [48]. 

Herein, shark gelatin obtained from waste industrial fisheries were used to form Ionogels at fixed gelatin concentration and different water/FIL ratio to design novel drug delivery systems with enhanced drug hydrophobic solubility in water in comparison to previous gelatin hydrogels [11,38] whilst retaining their performing mechanical properties and low cytotoxicity for its applicability as well as maintaining a drug controlled released. The hydrophobicity of the FIL, 1-ethyl-3-methylpyridinium perfluorobutanesulfonate employed as co-solvent in an aqueous media was selected to afford colloidal supramolecular gelatin complexes that will enhance the poor water solubility of therapeutic agents whilst avoiding to form a network that could disturb the gelatin network that provides the mechanical properties of the system.

Firstly, the nanostructure and miscibility of the generated water/FIL colloid systems upon increasing FIL concentration as well as its corresponding gelatin Ionogels will be correlated to the viscoelastic properties and solvation power of the selected drug model compound, Doxorubicin (DOX). Furthermore, the characterization of the afforded Ionogels by thermal, rheological, FT-IR, Raman, cryo-SEM, AFM and X-rays techniques will be reported to understand the effect of the dispersed FIL phase in the aqueous matrix within the global Ionogel structure as well as its role in the stabilization of both the gelatin and Doxorubicin [49,50]. 

In addition, release studies of two different antitumoral such as Doxorubicin and Mithramycin (MTM) from two different FIL content Ionogel networks were monitored to understand the molecular structure role on the dosage rate and compared to previous fish gelatin hydrogels drug delivery systems. 

## 2. Results and Discussion

**Figure 1:**
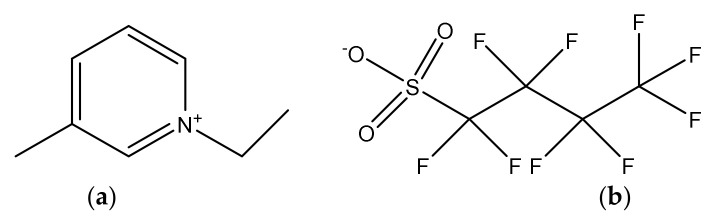
Molecular structure of 1-ethyl-3-methylpyridinium perfluorobutanesulfonate, [C_2_C_1_py][C_4_F_9_SO_3_]. (**a**) Cation 1-ethyl-3-methylpyridinium; (**b**) Anion perfluorobutanesulfonate.

Herein, a harmless fluorinated aprotic polar ionic liquid was selected to be mixed with water to afford a nanostructured liquid–liquid colloidal continuous phase [51], emulsion, to be used to both increase the drug solubility as well as affect the gelatin secondary structure [52] and thus, the hydrogel network. Likewise, the molecular structure of 1-ethyl-3-methylpyridinium perfluorobutanesulfonate (IL, Figure 1) impedes the formation of hydrogen bonds while promoting the water solubility of hydrophobic compounds and possessing compatible thermal and viscosity properties for both storage and application conditions [53].

Likewise, the different hydrophobic nature of the ionic liquids determines their dissolution in aqueous media compared to conventional salts, which are normally governed by the lattice energy and ion solvation [54].

The emulsion stability of the IL-Water system as well as its nanostructure of a series of different weight ratios of FIL/Water emulsions (Table 1) was firstly probed to correlate it with their respective gelatin Ionogels (Table 2) to understand their physicochemical properties and compared with the single components, i.e., the gelatin hydrogel and pure ionic liquid for the sake of clearness. The IL/Water ratio was maintained at higher water concentrations to avoid the inverse system as well as to retain the biocompatibility of the final Ionogels within the identical aggregate regime as found previously [51]. Besides, the pH of the IL/Water emulsions between 7.44–7.51 (Table S1) meets the standard body tissues, which is key for its biomedical application [55]. 

### 2.1. Emulsions Analysis

#### 2.1.1. Emulsions Stability

The self-aggregation behavior of the IL within the water continuous phase was initially probed by dynamic light scattering (DLS) to ascertain the generation of colloidal emulsions. The emulsion nanostructure revealed by DLS showed the presence of FIL aggregates with fairly constant dimensions throughout all the explored concentrations of around 4–6 nm (Table 3), however, a slighter larger aggregate at higher concentrations with a 4.8 and 6 nm in diameter was found for the 15IL/Water and 25IL/Water respectively in agreement with previous results in the same systems as well as analogous FILs [51] at 25 °C (Table 3). 

The stability of the emulsion was assessed by Zeta potential [56] by relating the interphase between the continuous phase and dispersed IL clusters. The Zeta potentials of IL/Water colloidal aggregates were found to diminish to −13 mV in contrast to the single IL −128 mV, suggesting a slight negative charge on the colloidal emulsion surface which is insufficient to repeal each other and prevent their aggregation as the absolute values are found below the stability threshold of 30 mV [57,58].

#### 2.1.2. Thermophysical Profile

The thermal transitions of the IL/Water emulsions have been monitored by DSC to ascertain if the structure of the IL aggregates dispersed in the aqueous phase influence the crystallization behavior of the single components such as the water and IL and to probe then their likely interactions. 

Likewise, the thermal analyses of the single components were firstly conducted at 1 °C/min to be compared to the corresponding emulsions. The DSC thermogram cooling cycle of the [C_2_C_1_py][C_4_F_9_SO_3_] ionic liquid (Figure S1) is characterized by several exothermic transitions in the thermal range from −48 °C to −23 °C related to its polymorphic crystalline structure (Figure S1) whiles the heating cycle thermogram reveals the melting of the crystalline domains at −23 °C and 5 °C, in good agreement with previous experiments [59,60,61]. The polymorphic nature manifested by the FIL structural domains such as polar, polar fluorinated, and apolar fluorinated is manifested by the characterized distinct crystalline phases at different temperatures [59]. Besides, the cooling thermogram of water shows that the crystallization occurs at around −18 °C while the melting onset proceeds at around 1 °C as shown in the heating thermogram (Figure S2d).

**Figure 2:**
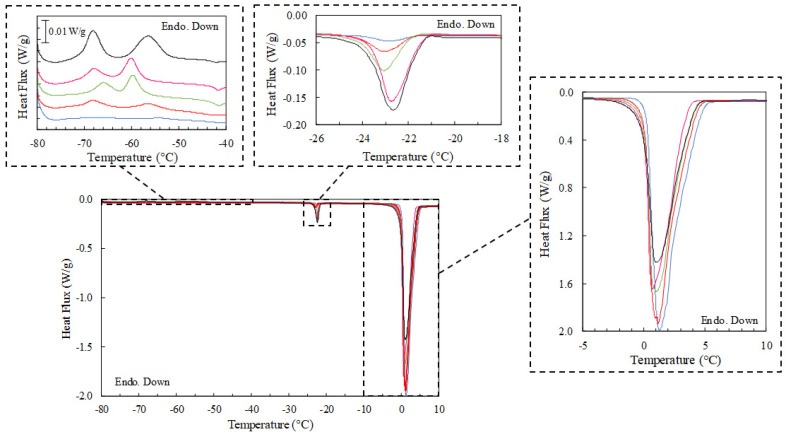
Heating DSC thermograms were obtained for the different IL/Water emulsions at 1 °C/min. (**—**) 1IL/Water, (**—**) 5IL/Water, (**—**) 10IL/Water, (**—**) 15IL/Water, (**—**) 25IL/Water.

The MDSC of the IL/Water emulsions was performed due to its higher sensibility to resolve the complex thermal behavior (Figure S3). The thermogram of the cooling cycle exhibit a shift of the crystallization water temperature to higher temperatures, suggesting the nucleating effect of the FIL aggregates. In addition, the crystallization onset of the FIL moved slightly whereas the crystallization rate was found to decrease dramatically up to feature the highest enthalpy change in the thermal range between −75 °C and −50 °C, which could be affected by the slower mobility within the frozen continuous phase. Furthermore, the heating cycle manifested the cold-crystallization within the same temperature range, confirming the slower crystallization kinetics. Furthermore, the enthalpies of the FIL crystallization follow a correlation with its weight fraction within the IL/Water emulsions even at the lowest concentration of 1IL/Water (Table 4, 1IL/Water and 1.6 J/g and 6.6 J/g for the 25 IL/Water and Figure S3), suggesting the packing of their molecules within the aggregates due to the lack of water molecules within the dispersed phase and the lack of interactions between the water and the FIL. In addition, the thermal overlapping of the melting of both FIL and water (0 °C and 5 °C, Figure 2 and Figure S2) complicates their identification even if is primarily attributed to the melting of water due to the proportional trend of the enthalpy with the water content in the emulsion. 

#### 2.1.3. Density and Dynamic Viscosity

**Figure 3:**
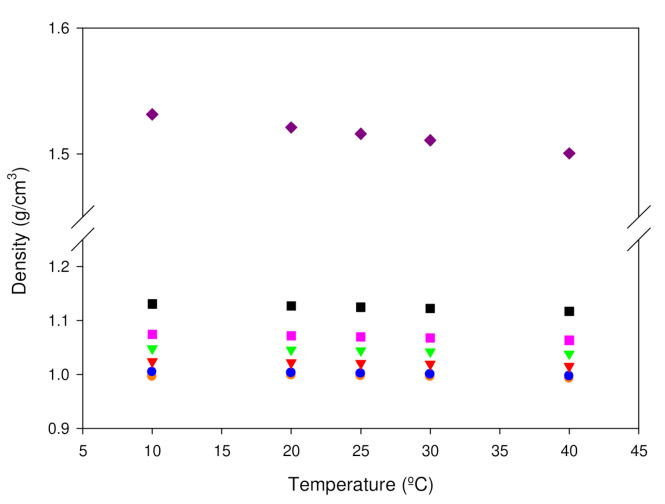
Density versus temperature of (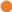) Water, (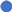) 1IL/Water, (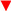) 5IL/Water, (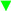) 10IL/Water, (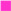) 15IL/Water, (⯀) 25IL/Water and (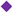) IL.

The density, *ρ*, of the single components i.e., water and the ionic liquid, as well as of the IL/Water emulsions was determined (Figure 3 and Table S2) at the working temperatures (storage and application of drug delivery systems) to understand the thermal stability of the formed FIL aggregates for all the emulsion compositions. The density of the Ionic liquid possesses 51–53% higher than the water. Thus, as expected, the density of the emulsions increases as the concentration of the ionic liquid rises. In addition, the density of the IL/Water emulsions remains fairly constant within the temperature range under study, which suggests the FIL cluster persistence [43,44].

**Figure 4:**
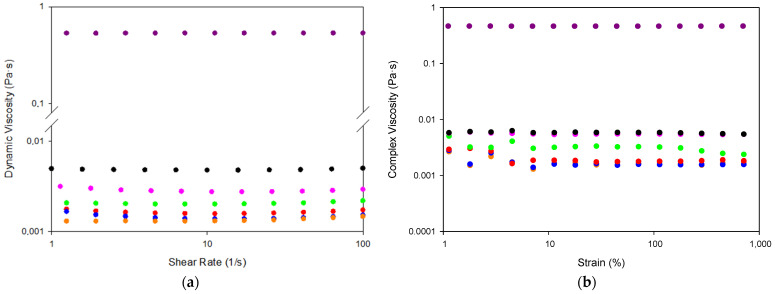
(**a**) Shear rate–shear viscosity flow curves and; (**b**) strain amplitude sweeps at a frequency of 0.05 Hz obtained for water, IL and IL/Water emulsions at 10 °C (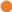) Water, (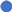) 1IL/Water, (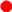) 5IL/Water, (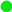) 10IL/Water, (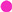) 15IL/Water, (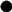) 25IL/Water and (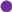) IL.

The existence of a network at all the IL/Water compositions formed by the FIL clusters which would percolate the continuous phase was evaluated by determining both dynamic viscosity (μ) and the complex viscosity and compared to that of water and neat (Figure 4). Likewise, the viscosity and in particular the dynamic viscosity will asses the origin of fluid resistance to flow either by intermolecular forces between water and FIL such as hydrogen bonding, short-range van der Waals interactions, and long-range electrostatic force or mainly the colliding of molecules [62,63].

The dynamic viscosities assessed at shear rates of 1–100 s^−1^ and a temperature of 10 °C (Figure 3a) show a Newtonian behavior. The dynamic viscosity of water was found to be 1.3 mPa·s in good agreement with the literature [64], whereas the dynamic viscosity of the FIL was determined to be 530 mPa·s in line with previous measurements [63].

The water emulsions dynamic viscosity shows a concentration dependence (4.8 mPa·s for 25 IL/Water, 3.3 mPa·s for 15 IL/Water, 2.5 mPa·s for 10 IL/Water, 2.0 mPa·s for 5 IL/Water and mPa·s for 1 IL/Water), however, fairly close to the water for all the systems that suggest the lack of a percolated network. In addition, the similarity of the complex viscosity to the dynamic viscosity modulus indicates the dominance of the loss component that confirms the absence of the cluster network.

#### 2.1.4. SAXS

**Figure 5:**
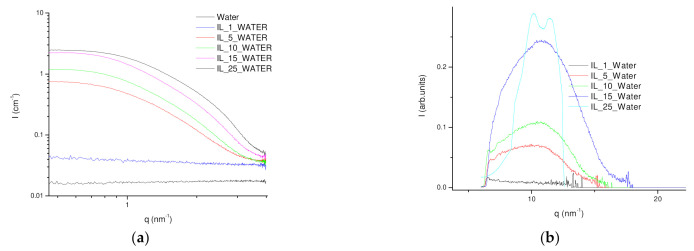
(**a**) SAXS and (**b**) WAXS profiles at room temperature (22 °C) of (**—**) Water, (**—**) 1IL/Water, (**—**) 5IL/Water, (**—**) 10IL/Water, (**—**) 15IL/Water and (**—**) 25IL/Water.

Besides, the nanostructure of the IL/Water emulsions was investigated by SAXS to understand both the dimensions and shape of the afforded aggregated as well as to ascertain the formation of a percolated network (Figure 5). The hydrophobic nature of the FIL was found to drive it to self-aggregate in water at low concentrations and with different concentration regimes [51]. The nanostructure of FIL cluster was found within the first aggregation regime to maintain fairly homogenous dimensions and shape factor upon the increase in concentration and only the rise of scatters is observed. The experimental SAXS profiles were fitted to the weakly correlated nanoscale mass fractal aggregates model in which aggregate size is described by R_g_ which is characterized by fractal dimension D_f_ and a correlation length. The dimension of the aggregates exhibits a steady R_g_ of ca. 1.7 nm up to 25IL/Water, in which a slight reduction to 1.55 nm was found (Table S2) and is in fair agreement with the dimensions obtained by DLS. In addition, the fractal dimension was found to increase from 3.3 to 4 while the correlation length was reduced from 2.99 to 2.34 as the FIL concentration increased (Table S2). Furthermore, the WAXS analysis of IL/Water emulsions has proved the packing of the FIL molecules as its concentration increases within the aggregates with a periodicity distance of around 5.4 Å which suggests aromatic stacking.

The nature of the interaction between FIL aggregates in water was probed by Raman spectroscopy. The inherent complexity of the interactions among the ionic liquid molecules itself and with the continuous aqueous phase drives the clustering of the IL even at low concentrations. The lack of IL molecular groups with hydrogen bonding donors or acceptors in its molecular structure together with the hydrophobic character of both cation and anion should reduce the solvation shell. The Raman spectra of [C_2_C_1_py][C_4_F_9_SO_3_]/water (Figure S4) show a continuous intensity increase within the IL/Water emulsions as the FIL concentration increases apart from minor variations of he CH stretching vibration of the pyridinium cation suggesting the highly hydrophobic character that drives the aggregation [65].

**Figure 6:**
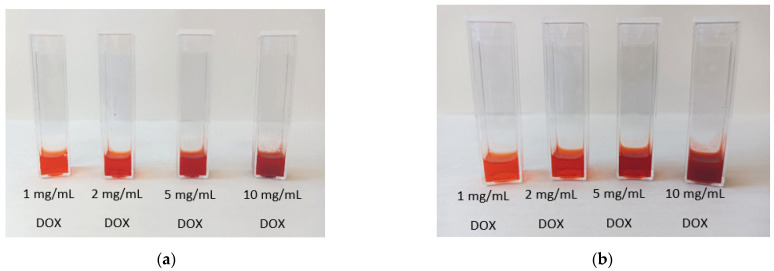
Images of the Doxorubicin-loaded IL/Water emulsions prepared at (**a**) 10IL/Water, and (**b**) 25IL/Water.

In addition, the enhancement of the poor Doxorubicin solubility in aqueous media by the IL/Water emulsions was qualitatively evaluated at two different FIL contents to understand its role at room temperature by visual assessment of a homogeneous solution. A significant increase in drug load in aqueous media by the IL/Water emulsions was confirmed as the water Doxorubicin solubility, 2.6 mg/mL at 25 °C [66], was enhanced up to 5mg/mL in the 1IL/Water and to at least 10mg/mL for 25IL/Water system(Figure 6). 

**Figure 7:**
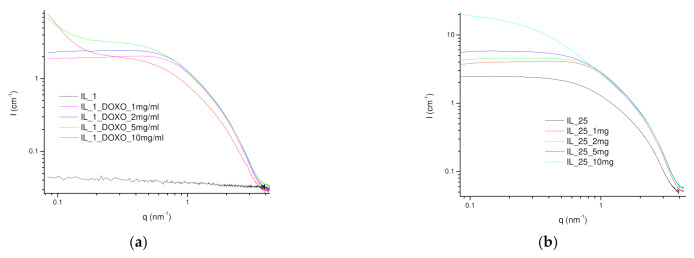
(**a**) SAXS profiles at room temperature (22 °C) of (**a**) 1IL/Water loaded with DOX and (**b**) 25IL/Water loaded with DOX.

The nanostructure of the Doxorubicin-loaded IL/Water emulsions probed by SAXS has exhibited to retain fairly in line with the corresponding IL/Water emulsion the shape factor and dimension of the formed aggregates (Figure 7). Similarly, the SAXS experimental intensity was adjusted to the weakly correlated nanoscale mass fractal aggregate model to extract the structural parameters (Table S3) yielding a slight increase in the R_g_ of 1.7 nm for the 25IL/Water DOX loaded with a similar correlation length of 2.3 and a decreased fractal dimension to 3.75 as the DOX concentration was augmented, evoking their presence on the aggregates. However, the emulsion with higher DOX content (25IL/Water/DOX-10mg/mL) was fitted with a heterogeneous sphere with a fractal structure that indicates the formation of spherical-like aggregates with smaller dimensions and fairly shorter correlation length (R_g_ of 1, 𝜉 of 1.6, Table S3), suggesting its participation to the dispersed interconnected phase. In addition, the chemometric analysis of the Raman spectra of the 25IL/Water DOX loaded (Figure S5) has mainly revealed a negative correlation between the DOX and FIL vibrational bands that described the 99.3% variance of the spectra (PC1) suggesting a weak interaction through electrostatic forces between both molecules. 

The gelation of ILs induced by the formation of composites with either polymers or other inorganic material yields the so-called Ionogels that combine the properties of their constituents, enhancing in particular their mechanical resistance [67]. Ionogels formed by gelatin and harmless IL are ideal candidates to generate drug delivery systems as a result of their featured multifunctionality and their synergetic effect in new drug development [27]. The effect of IL at an identical concentration of the previously investigated emulsions on the gelatin hydrogel structure (Table 2) was evaluated to be correlated with the drug uptake and release mechanism.

### 2.2. Ionogels Analysis

#### 2.2.1. Electrophoresis

The influence of IL/Water emulsion on the solubility of the heterogeneous gelatin chain architecture was characterized by Sodium Dodecyl Sulfate Polyacrylamide Gel Electrophoresis (SDS-PAGE) and thermogravimetric analysis (TGA). Likewise, the featured broad weight distribution and rich chain conformation with several secondary structures of the fish gelatine could stabilize different chain structures due to the interaction with the apolar amphiphilic IL aggregates compared to the simple hydrogel [52]. In addition, the structural variability from gelatin batch to batch could be homogenized.

**Figure 8:**
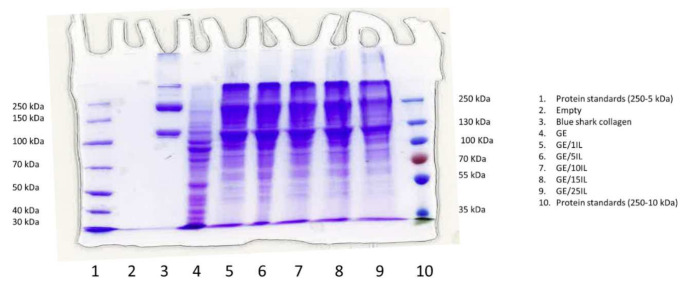
SDS-PAGE of Blue shark collagen and Ionogels with increasing concentration of ionic liquid.

Electrophoresis gel manifested the differences in molecular weight of gelatin dissolved simply in water in contrast to the gelatin in the ionic liquid emulsions (Figure 8) and for the sake of clearness compared to separated collagen extracted from blue shark skin (lane 3). The two major bands found for collagen correspond to single α chains of Mw slightly above 100 kDa, and β chains of around 200 kDa that consist of dimers of covalently linked α chains. In addition, less intense bands appear at longer migration times, possible due to γ chains (trimers of α chains) and other supramolecular aggregates. In contrast to collagen, species of molecular weight below 100 kDa predominate in gelatine dissolved in water (lane 4), although less intense bands of α and β chains, and even higher molecular weight fragments, are also visible which are typical of gelatin chain distribution complexity.

Remarkably, the gelatin electrophoresis gel is characterized by the migration of a great number of fragments of different sizes that produce a continuous distribution in contrast to collagen where each band is clearly defined. The collagen fragmentation during gelatin extraction produces the multiple electrophoresis bands related to the disruption of inter- and intramolecular ligands of the collagen and peptides. Likewise, the gelatin obtained consists of a blend of molecular segments featuring a molecular weight between 80 to 250 kDa [68], although aggregates with a higher molecular weight might also persist in the gelatin composition.

Upon increase in the ionic liquid for the Ionogels, (lanes 5–9, Figure 8) the intensity of the gelatin bands, initially concentrated below 100 kDa in aqueous media (lane 4), clearly shifts to higher molecular weights. Particularly interesting are the intense bands that appear at migrating distances similar to those of collagen, suggesting the stabilization and rearrangement of gelatin chains into complexes of similar molecular weight to those of α and β chains, and possibly also γ chains and other supramolecular structures observed in collagen.

#### 2.2.2. Thermophysical Profile

**Figure 9:**
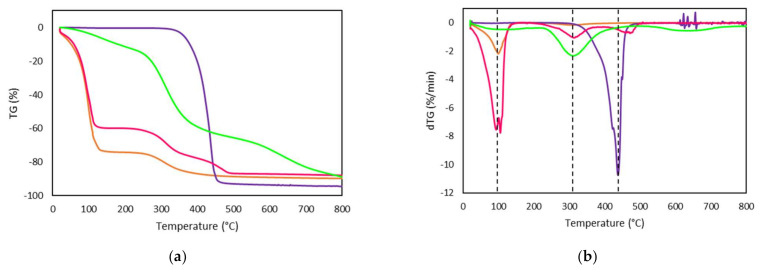
(**a**) TGA thermogram; (**b**) dTGA thermogram obtained from TG curves of (**—**) Dry GE, (**—**) GE, (**—**) GE/15IL and (**—**) IL.

The thermal stability of the constituents of the Ionogel, as well as qualitative chain architecture analysis, was conducted by TGA in the GE/15IL system as an example for the rest of the Ionogels. The TGA analysis (Figure 9a,b) confirms that the system was thermally stable up to ca. 80 °C which is in line with the Ionogel preparation methodology as well as for the pharmaceutical application conditions. The thermograms of the Ionogels as well as with their constituents and in particular for the gelatin batch employed for the current work together with its hydrogel (GE) were analysed by their respective derivative (dTGA) for the shake of clearness (Figure 9b and Table 5). Similarly, to previous gelatin batches [38], the gelatin degradation profile features firstly a step correlated with the different forms of associated water with each nanostructure. Likewise, a weight loss of 12% at 89 °C was found for dry gelatin and with an obvious higher weight loss of around 70% for both the gelatin hydrogel and gelatin Ionogel related to water. 

The next weight loss stage around 300 °C is due to the decomposition of the gelatin chain [69] as a result of the cleavage of gelatin peptide bonds of low molecular weight protein fragments and structurally bound water. The increase in decomposed gelatin at a higher temperature for the Ionogels compared to the gelatin hydrogel, suggests the stabilization of higher Mw gelatin molecules thanks to the FIL [70]. In addition, the FIL is characterized by a single weight loss step which accounts for 95% that occurs at around 437 °C. In addition, Ionogel also possesses a weight loss related to the ionic liquid of 12% although at a higher temperature of 479 °C, indicating that FIL interacts with the gelatin chain molecules [71] and in particular, with the higher molecular weight in agreement with the electrophoresis gel results.

#### 2.2.3. Spectroscopy Analyses

**Figure 10:**
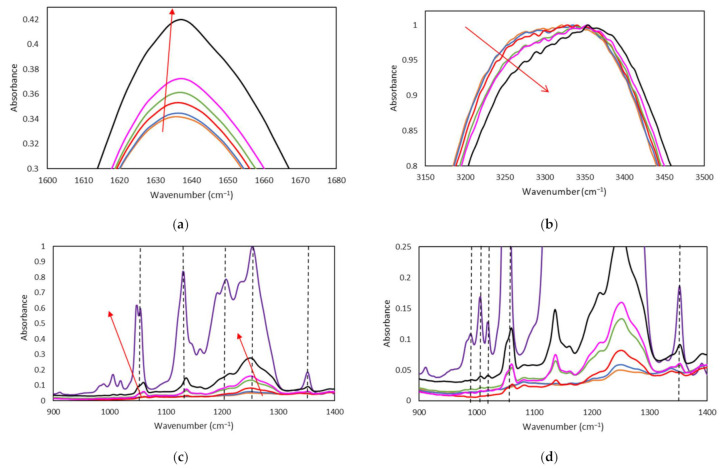
FTIR spectra of the Ionogels. Secondary structure 1600–1700 cm^−1^ region (**a**), 3150–3500 cm^−1^ region; (**b**) 900–1400 cm^−1^ region; (**c**,**d**) of: (**—**) GE, (**—**) GE/1IL, (**—**) GE/5IL, (**—**) GE/10IL, (**—**) GE/15IL, (**—**) GE/25IL and (**—**) IL.

**Figure 11:**
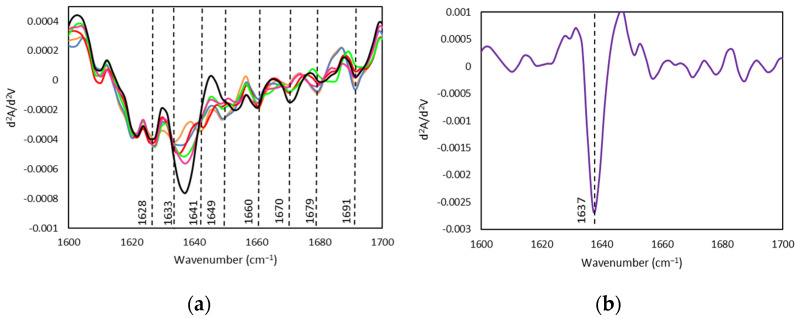
Second Derivative, FTIR spectra of the Ionogels. (**a**) Secondary structure 1600–1700 cm^−1^ region of (**—**) GE, (**—**) GE/1IL, (**—**) GE/5IL, (**—**) GE/10IL, (**—**) GE/15IL,(**—**) GE/25IL and (**b**) (**—**) IL.

FTIR spectroscopy analyses have been conducted to characterize the effect of the FIL on the secondary structure of gelatin-based Ionogels (Figure 10). However, the expected increase in the FIL bands as its concentration increase could be potentially accompanied by shifts on the gelatin spectra to recognise likely interactions, although the identification of the single components elements is ambiguous due to the overlapping of their vibrational bands. The FIL is dispersed on the medium as support that could facilitate multiple interactions with the gelatin such as ionic, hydrogen, van der Waals, etc. which result in a supramolecular network that will influence the conformation of the protein [72]. Specially, the gelatin nanostructure footprint by spectroscopy is related to the vibrational bands of the amide A (3600–2300 cm^−1^), amide I (1700–1600 cm^−1^), amide II (1500–1560 cm^−1^), and amide III (1200–1300 cm^−1^). Likewise, the analysis of the second derivative to amide band I facilitate the extraction of the rich detailed signals about the secondary structure of the gelatin (Figure 11, Table 6) [73,74] in good agreement with other fish [75].

Similarly, the FTIR vibration bands related to the FIL, [C_2_C_1_py][C_4_F_9_SO_3_] appeared at 1508 cm^−1^ for the pyridinium ring (C=N) while the band at 1055 cm^−1^ corresponded to the SO_3_ and the bands at 1207–1253 cm^−1^ and 1131 cm^−1^ are associated to the stretching of CF_2_ group (Table S4).

Of particular interest for the secondary structure is the C=O stretching vibration band which originates from the amide groups associated with in-plane NH bending and CN stretching modes [37] as its frequency depends on the hydrogen bonds related to the C=O and NH functional groups.

The amide A band that arises from the stretching vibration of the N-H group of the peptide appeared at 3321 cm^−1^ for GE and gradually increased with the addition of FIL until 3353 cm^−1^ (Figure 10b), indicating the reduction of the NH bond distance The stability of higher molecular weight gelatin chains could explain the shortening of NH bond as might interact through hydrogen bonds between the N-H moiety with the peptide chain or associated with electrostatic interactions with the FIL due to the lack of hydrogen bonding donors/acceptors in its molecular structure. Furthermore, the Ionogels formulations spectra exhibited the vibration band at 1649 cm^−1^ which is indicative of the random coil conformation, although slightly decreases upon FIL content increase (Figure 11). In addition, the changes observed in both the β-turns and β-sheet of the polypeptide backbone are believed to promote higher gelatin stability [76] due to the slightly more compact three-dimensional produced by the interaction with the IL. Furthermore, the enhancement of the amide II band at 1550 cm ^−1^ together with the appearance of the vibrational band at ca. 1650 cm ^−1^ (Figure S6, Table S5), as well as the shorter spectral distance of 100 cm^−1^ between the amide I and II [77] suggest the enhancement of the triple helix content as the FIL content increases in the Ionogels whereas the band at 1633 cm^−1^ was attributed to the water bound peptides of denatured collagen. Besides, the hydrophobic nature of the ionic liquid would interact mainly with the hydrophobic regions of the gelatin. Likewise, the stretching vibrational modes of the C-F bonds of the CF_2_ and CF_3_ groups that usually appear in the spectral range between 1000 and 1300 cm^−1^ were found to increase the wavenumber for the Ionogels gelatin as the FIL concentration increases (1 and 5% wt not observable) that suggest an interaction between the fluorinated part of the ionic liquid with the aliphatic gelatin segment [78,79].

Similarly, the principal component analysis (PCA) of the Raman spectra Ionogels has revealed that indeed, the main variable that explains the spectral variance is the FIL concentration (ca 79%), however, the PC2 (13%) confirms the change in the amide region (Figure S7) related to the gelatin stabilization by FIL.

#### 2.2.4. Phase Change Properties

In addition, the thermodynamic behavior of the Ionogels was probed by DSC together with the dry gelatin, as well as gelatin hydrogel GE DSC to understand the parameters that govern the gelation mechanism related to the suggested by spectroscopy interactions between GE and FIL and its structural readjustment.

**Figure 12:**
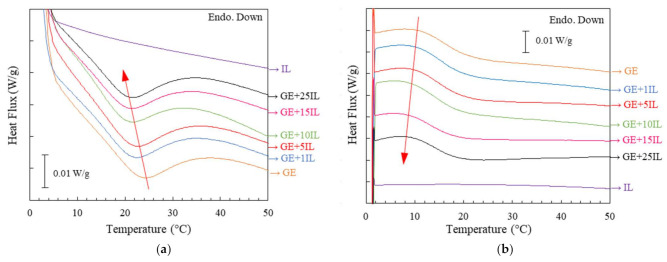
(**a**) Heating and (**b**) cooling thermograms of GE and Ionogels at 5 °C/min. (**—**) GE, (**—**) GE/1IL, (**—**) GE/5IL, (**—**) GE/10IL, (**—**) GE/15IL, (**—**) GE/25IL and (**—**) IL.

Similarly to the tuna gelatin [38] the dry shark gelatin is characterized by a glass transition observed around Tg ~ 62 °C (Figure S8) associated with the amorphous segments of the gelatin. Likewise, the reversible transition that occurs at around 23 °C for the GE shark gelatin hydrogel (Figure 12a,b) with an enthalpy of 4.2 J/g is generally attributed to the unfolding of gelatin randomly distributed throughout the hydrogel. Likewise, the disruption of the network of gelatin hydrogels is related to the unfolding of triple helices [80] whilst the so-called melting of the dry gelatin crystalline junctions results in the rupture of the lateral stacking of triple helices. The unfolding of gelatin shifts towards lower temperatures as the FIL concentration (gelatin concentration is fixed at 25% wt) is increased while the area of the thermal event is reduced (Table 7).

Previously, the thermal modification of the gel/sol transition for both temperature and/or enthalpy of gelatin hydrogels doped with different salts was correlated to affecting the triple helix that affects the coil-helix transition [80]. Likewise, a decrease in triple-helix content is indicated by the diminishing of the associated area/enthalpy whilst the temperature shift is considered to be correlated with the length of the triple helix as the energy required to disrupt the hydrogel structure is related to the number of junction points [80]. In particular, salts featuring low hydrated anions were found to be able to interact with the amide moieties of the amino acids within the gelatin chain that disrupt the hydrogen bonding of the polypeptides as well as the electrostatic interactions between the protonated amines of the positively charged side chain peptides of the gelatin which depend on the salt concentration [80]. The pH of the gelatin Ionogel throughout the concentration remained around 7.5, suggesting either a weakly charged electrolyte gelatin behavior or neutral, although locally dipole-dipole interactions are expected to occur.

In addition, the energy reduction associated with the hydrogel disruption was potentially attributed to a minor conformational of the tertiary and secondary gelatin structures produced by the interaction with the FIL.

**Figure 13:**
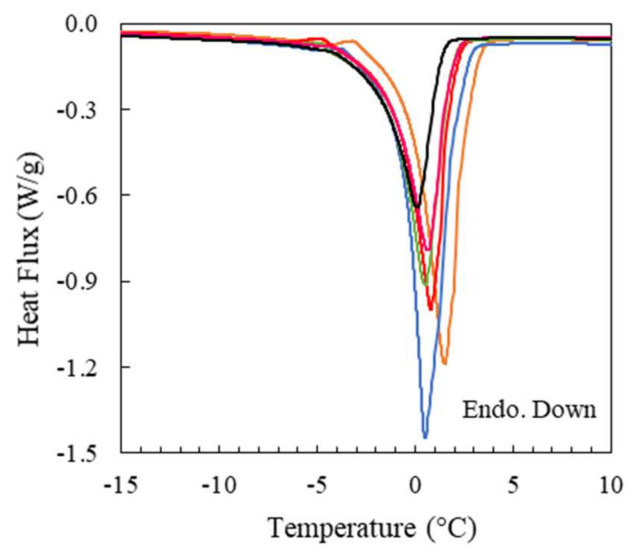
Heating thermograms of GE and Ionogels at 1 °C/min: (**—**) GE, (**—**) GE/1IL, (**—**) GE/5IL, (**—**) GE/10IL, (**—**) GE/15IL and (**—**) GE/25IL.

Besides, two endothermic peaks are observed in the GE thermogram in the heating ramp at 1 °C/min (Figure 13), that were previously related to either melting of the water most closely bound to the gelatin structure (−4 °C) and the melting of the “free water” at around −0.2 °C for tuna gelatin hydrogel [38] that tend to vanish as the FIL concentration is increased. Furthermore, the addition of the IL within the gelatin hydrogel produced a freezing point depression (Table S6) and the subsequent shifting of the melting point of the water towards lower temperatures (onset temperature of around −0.8 °C). The modification of the water crystallization mechanism by increasing the FIL concentration in the Ionogels suggests the solubilization of gelatin chain as the FIL is increased, which in turn, yields them accessible to the water and thus, affects the chain mobility. Furthermore, the enthalpy associated with water melting also diminishes along with the enhancement of the concentration of IL, however, the lack of linear dependence with the water concentration indicates the absence of colligative properties, suggesting the solute-solute interactions (gelatin-gelatin).

**Figure 14:**
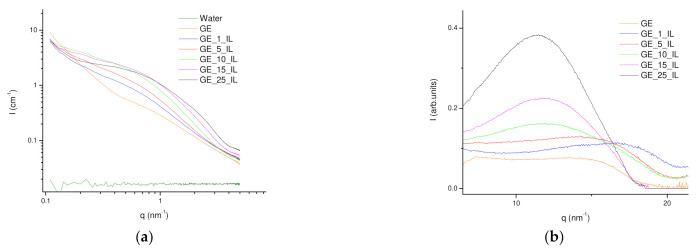
(**a**) SAXS and; (**b**) WAXS profiles of the Ionogels at different concentrations.

The nanostructure of Ionogels was revealed by simultaneous SAXS/WAXS experiments to ascertain the quasi semicrystalline structure of gelatin that was found to retain typical triple helix crystalline domains depending on the conditions with the fibrillar network that percolates the volume. The SAXS profiles of the Ionogels were in agreement with the gelatin hydrogel structure that was previously modelled to the heterogeneous sphere with fractal structure [11]. Likewise, the aggregates feature equivalent dimension with an R_g_ of ca 1nm associated with the fractal size, although the correlation length increased upon FIL content up to 15% as at GE/25IL the correlation length was reduced equivalent to the GE hydrogel distance but with a much higher fractal dimension that confirms the higher compactness of the system. In addition, the fitting of the SAXS profiles at temperatures over the folding temperature revealed by DSC showed an increase in the correlation length. Besides, the local packing of the Ionogels has shown the persistence of the FIL packing (Figure 14b, Table S7) although with a higher distribution that indicates the presence of the FIL aggregates within the Ionogel nanostructure.

#### 2.2.5. Morphological Analysis

In addition, the microstructure of the afforded Ionogels was probed in the real space by cryo-SEM as well as by AFM to correlate it with the mechanical and topological release properties, however, the experimental limitations related to the prior etching step involved in the sample preparation for the cryo-SEM [81] and to the complex topography with frequent empty spaces for the AFM influence their realistic representation.

**Figure 15:**
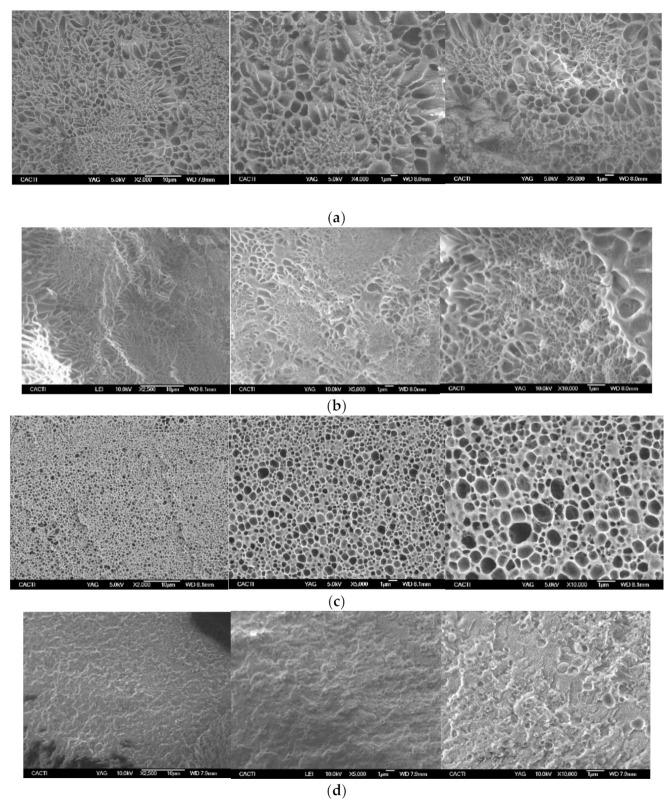
Cryo-scanning electron microscopy of hydrogel gelatin and Ionogels: (**a**) GE; (**b**) GE/5IL; (**c**) GE/15IL; (**d**) GE/25IL.

Likewise, the darker areas of the SEM micrograph are related to the water that was not subjected to the sublimation whilst the lighter objects correspond to the gelatin after etching. The shark gelatin exhibits a sponge/coral-like morphology [11] equivalent to other fish hydrogels as tune gelatin [38] which are typical of adequate gelling properties (Figure S9). Similarly, the Ionogels exhibit a morphological network of heterogeneous porous that tend to be homogenized both in their dimensions and distribution as the FIL concentration is augmented (Figure 15 and Figure S9), suggesting its templating effect and homogenous distribution in agreement with the effect on the morphology of IL in gelatin films where IL aggregates were found [82], however, experimental effects related to differences on the etching step cannot be discarded. In addition, the apparent reduction of pore size observed in by the quantitative analysis (Figures S10 and S11) as the FIL content is increased up to a uniformed morphology is attained at 25% wt of FIL (GE/25IL), suggest that FIL is able to distribute the gelatin chains due to their stabilization in solution.

**Figure 16:**
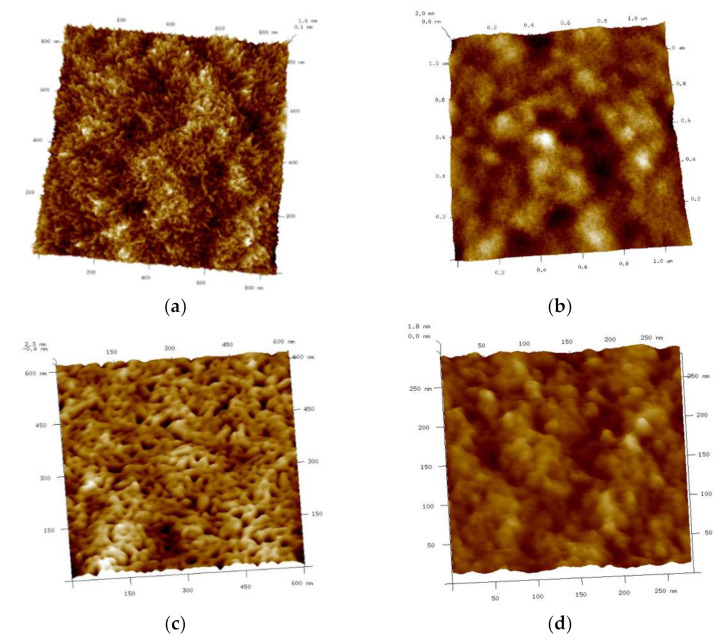
AFM images 3D of shark gelatin hydrogel.; (**a**) Zone 1 with a field of view of 1.6nm; (**b**) Zone 2 with a field of View 2nm; (**c**) GE/5IL with a field of view 600 × 600nm^2^; (**d**) GE/25IL field of view 260 × 260nm^2^.

Furthermore, the structural analysis of the Ionogels surface was studied by AFM as they represent the morphological aspects without sample preparation artefacts. The GE hydrogel has shown different roughness areas where a continuous surface is observed, with similar height (brown colour), without significant cavities, and a few protrusions (yellow colour) (Zone 1, Figure 16a,b and Figure S12a,c), although exhibiting a coacervate structure without defined geometric shape typical of porous materials. In addition, a greater height variability with aggregates with spherical-like objects was also found across the hydrogel surface that depicts the heterogeneous porous shape and dimensions (Zone 2, Figure 16c,d and Figure S12b,d) which is persistent at higher magnification (Figures S12d and S13) and in good agreement with cryo-SEM micrographs at comparable magnification (Figure S14).

Similarly, the morphological analysis by cryo-SEM of the Ionogels at two different FIL contents (5% and 25%) as representative of the system has confirmed to retain the structural porosity upon FIL addition (Figure 15b–d and Figure S9c–f).

However, the Ionogels topography exhibit more homogenously distributed pores and smaller dimensions as the FIL concentration is increased (Figure 16c,d and Figure S15a,c,d). Likewise, the roughness parameters [60] for both GE hydrogel (Table 8) and its corresponding Ionogels (Table 9) have shown fairly variability in the surface texture upon FIL addition. The quantitative analysis of the roughness as the average roughness (Ra) and root mean square surface roughness (Rq) together with its height surface symmetry and sharpness distribution (the surface skewness and kurtosis), highlighted the increase in roughness with increasing porosity and peaked morphology although, the image processing methodology could be influenced their assessment.

Furthermore, the quantitive roughness analysis was also performed by profilometer by WLOP technique (Figure S16) and has shown inconsistent surface parameters compared to AFM (Table S9) likely due to the greater field of vision that increases the probability of finding larger topographic features, however, the morphological change upon FIL content increase was found persistent in both analysis.

#### 2.2.6. Mechanical Properties

In addition, the mechanical properties of Ionogels were assessed by rheological measurements to both characterize the system structure as well as to evaluate the hydrogel storage and administration performance.

Likewise, the network stability of the Ionogel was probed to define the linear viscoelastic region (LVR) by subjecting the hydrogels to deformation sweeps at 10 °C which is just above the folding transition observed by DSC.

**Figure 17:**
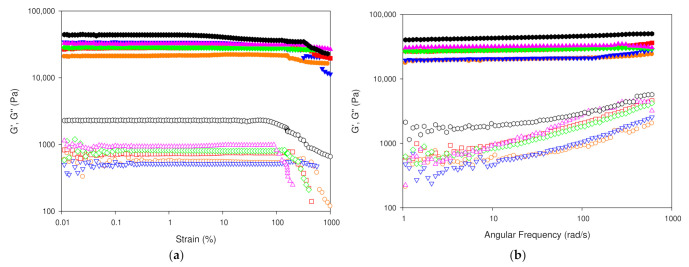
Store (G′) and loss (G″) moduli (**a**) versus strain; (**b**) versus angular frequency for: GE and GE/IL hydrogels: (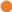) GE, (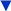) GE/1IL, (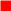) GE/5IL, (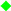) GE/10IL, (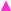) GE/15IL and (🔴) GE/25IL.

The storage modulus (G′) and the critical strain at which the deformation stress will produce, irreversibly, the deformation of gel structure, which were found to be higher for the Ionogels compared to the GE (Figure 17a). The marked increase in both G′ and the critical stress for the Ionogels suggests the formation of an enhanced entangled fibrillar network that is able to immobilize gelatin fibres and thus, the increased resistance to flow [83].

However, the higher increase in G″ by an order of magnitude compared to slighter both enhancement of the G′ and smaller LVR in contrast to GE, suggest the formation of a double percolated network in the Ionogels. Furthermore, the temperature sweep of the Ionogels probed the presence of a weaker gel phase above the folding transition.

Besides, frequency sweeps of both the gelatin hydrogel and Ionogels were performed to understand the time-dependent viscoelastic behavior of the gel networks. The elastic component predominated over the viscous in the entire region of linear viscoelasticity for the gelatin hydrogel as well as for the Ionogels (Figure 17b). Furthermore, the flow behavior of the Ionogel, indicates the formation of a large enhancing of the elastic network, being a characteristic of complex gels that approach a solid type trend. Both modules increased with the angular frequency and also with the addition of IL, showing a much higher loss and storage modulus value, indicating greater gel stability.

**Figure 18:**
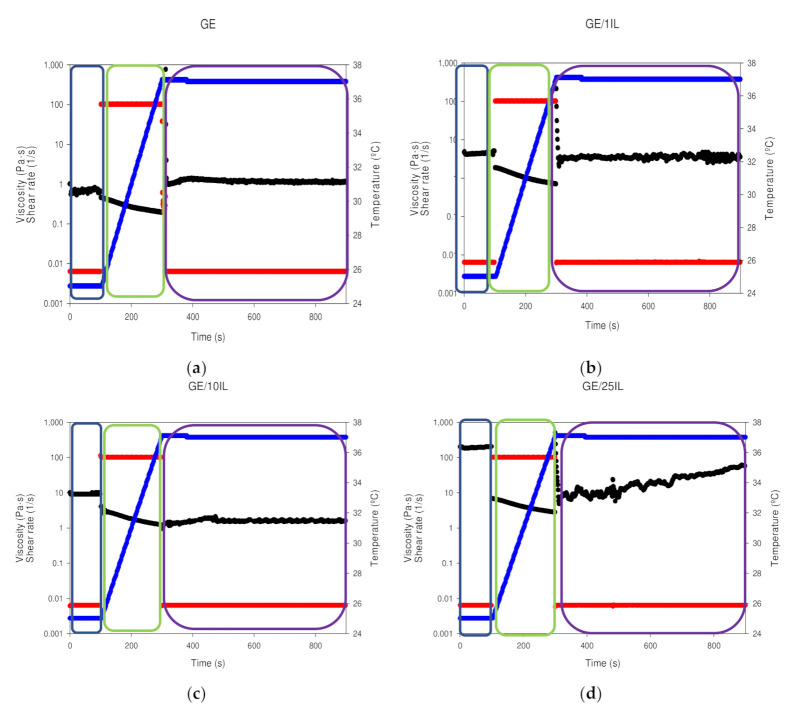
Syringe test results of gelatin hydrogel (**a**), GE; (**b**), GE/1IL; (**c**) GE10IL and; (**d**) GE/25IL. Viscosity (black colour), shear rate (red colour), temperature (blue colour).

Besides, the assessment of the administration and storage performance of the Ionogels was conducted by the so-called *syringe test* [84] which mimics the mechanical behavior of Ionogels during a local administration through a needle by monitoring the apparent viscosity of the Ionogels at three different steps that simulate both the mechanical and thermal conditions. Initially, the storage capability was evaluated in the first step by an oscillatory sweep at a constant strain of 0.1% which is within the LVR. An increase in viscosity was found as the FIL concentration was increased within the Ionogels composition from 1.00 ± 0.09 Pa·s (*p* < 0.03) for GE (Figure 18a, blue box to 4.14 ± 0.35 Pa·s, 9.15 ± 0.36 Pa·s and 190.7 ± 6.5 Pa·s (*p* < 0.03) for GE/1IL, GE/10IL and GE/25IL, respectively (Figure 18b–d, blue box). Moreover, a gradual increase in the temperature to 37 °C at a constant shear rate of 100 s^−1^ (Figure 18a–d, green box) proceeded in the second step to reproduce the injection with a needle of 0.9 mm in diameter and the needle thermal gradient (from 25 to 37 °C) along its pathway to the human body. A decrease in viscosity below 5 Pa·s was found for GE and the corresponding Ionogels when the shear rate attained 100 s^−1^, yielding 0.3 ± 0.1 Pa·s, 1.2 ± 0.4 Pa·s, 1.9 ± 0.7 and 4.3 ± 1.3 Pa·s for GE, GE/1IL, GE/10IL and GE/25IL, respectively which are in the same order of magnitude to previous tuna gelatin hydrogels [38] and other batches shark gelatin hydrogels [11]. Furthermore, the viscosity of both Ionogels continuously decreased progressively as the temperature increased which proved the feasibility of the injection. The viscosity at the physiological conditions was replicated by applying a constant strain at 0.1% at 37 °C (Figure 18a–d, purple box) in the subsequent step, showing the decrease in the initial viscosity for the Ionogels with higher FIL content that in turns, gradually increase along time to recover the start viscosity with viscosity just after the injection analogous to the human liver, 1.2 ± 0.2 Pa·s, 3.4 ± 0.5 Pa·s, 1.8 ± 0.4 and 22.4 ± 14.1 Pa·s for GE, GE/1IL, GE/10IL and GE/25IL, respectively. The slow increase in viscosity, for the Ionogels with higher FIL content, suggests the slower kinetics of the emulsion structure formation. In addition, the higher viscosity modulation featured by Ionogels with higher FIL content indicates the viability of intra-articular injection through a needle diameter of 0.9 mm that will possess a longer residence after the inoculation inside the human body due to its higher final viscosity.

#### 2.2.7. Drug release Experiments

**Figure 19:**
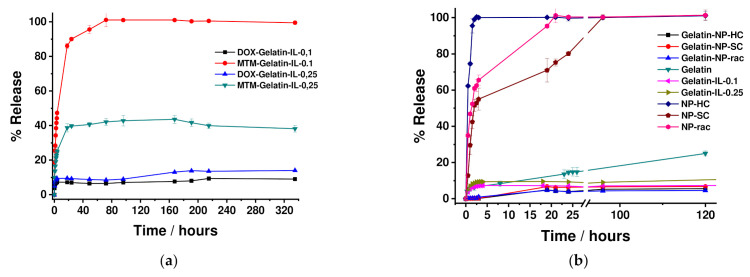
(**a**) DOX and MTM release from the GE/10IL and GE/25IL; (**b**) Comparison of the DOX release from different drug delivery systems, mainly based on gelatin hydrogels.

The release mechanism of both DOX and MTM antitumoral from the Ionogels of two different formulations with different FIL content such as GE/10IL and GE/25IL was followed by UV-vis (Figure 19a) to understand the likely interactions with the Ionogel nanostructure depending on the molecular structure of the drugs. The release profiles showed a rapid burst release in the first 5–6 h for both DOX and MTM at both Ionogels formulations. However, a very slow release that is independent of the Ionogel formulation is followed after the burst release for DOX which slightly increases after 120 h, suggesting a high affinity of DOX for the gelatin interface. Likewise, the Ionogel is able to load higher content of DOX as well as being capable of releasing it continuously over time, which is ideal for oral administration applications.

On the other hand, the release of MTM showed a typical triphasic profile, where the burst release is followed by a stage of diffusion through the water/ionic liquid interface between 5–20 h. Thereafter, the MTM release slowed down to reach a continuous step, although with a higher Ionogel formulation dependence. The MTM release rate was found faster for the Ionogel formulation with a lower content in the FIL system with a total release in 80 h for GE/10IL whilst the release is maintained at 40% even up to 330 h GE/25IL, which suggests a higher affinity of MTM for ionic liquid interfaces within the system.

The comparison of the different DOX releases from different drug vehicles mainly based on gelatin hydrogels highlights the importance to design nanocomposites with specific multifunctionalities for the customization of both the encapsulation and subsequent release of DOX [11,57] to achieve personalized medicine (Figure 19b). Likewise, the composition of the hybrid system enables to easily modulate of on-demand drug release profiles with different clinical translation pathways with similar mechanical properties required for the drug storage and its local administration.

## 3. Conclusions

The design of biocomposites to combine multiple specific functions is a promising approach to design novel drug carriers in straight forward fashion with enhanced therapeutic agent load and subsequent control release together with the mechanical properties required to be stored and administrated locally in the human body to achieve personalized pharmaceutical applications.

Previously, fish gelatin derived from waste fisheries was proved to be an ideal support for uptaking several antitumoral as well as fulfilling the viscoelastic properties to administrate intravenous therapeutic agents. In addition, a perfluorinated ionic liquid (FIL) with an aprotic polar character that was found to aggregate in aqueous media was identified to assist in the drug solubilization due to its amphiphilic nature. Ionogels formed from shark gelatin and the perfluorinated ionic liquid as co-hydrosolvent were generated to load two different antitumoral such as Doxorubicin and Mitramycin with a different molecular structure to assess their role in the release mechanism.

The nanostructure of the FIL/Water emulsions was found to feature weakly correlated nanoscale mass fractal aggregates that were slightly modified upon the addition of Doxorubicin. In addition, the structural characterization of the Ionogels has shown the persistence of heterogeneous aggregates with the fractal structure of the gelatin hydrogels, however, an increase in the correlation length was followed by the rise of the FIL content up to 25% of FIL composition was attained (GE/25IL) where the correlation length was reduced equivalent to the GE hydrogel distance although with higher compactness of the system. Furthermore, the microscopic morphology of the Ionogels was accordingly modified with respect to native gelatin hydrogel, although retaining the porous-like structure that controls both the mechanical properties and the topological drug release mechanism.

The storage and administration performance for local administration was assessed by the so-called “syringe test”, exhibiting similar storage and viscoelastic properties upon the injection through a needle of 0.9 mm even though a post-injection viscosity recovery was found upon FIL concentration increase compared to the single gelatin that suggests a longer residence time after the inoculation inside the human body. Likewise, the Ionogels were proved to greatly enhance the solubilization of DOX by incorporating it into the dispersed correlated fractal aggregates in the aqueous solution whilst maintaining the viscoelastic properties required to store and local administrate the therapeutic agents. Besides, the release profiles of both antitumoral have shown the preference interaction of the Mitramycin with FIL to control its dosage rate compared to Doxorubicin which is mainly regulated by its interaction with the gelatin hydrogel network.

## 4. Materials and Methods

### 4.1. Materials

Propegal S.L. has supplied the skin shark fish wastes (BS—blue shark, Prionace glauca) to produce the gelatin that was extracted in the laboratory of Recycling and Valorisation of Waste Materials (REVAL) of the Instituto de Investigacións Mariñas de Vigo (IIM) CSIC (Spanish National Research Council) in Vigo, Galicia, Spain [85].

The blue shark skin type was dispersed in distilled water and heated slowly up to 60 °C to then cooled to room temperature (22 °C) to produce a gel upon cooling. The selected weight of Shark gelatin was measured by a Mettler AE-240 electronic balance, with an accuracy of 5·10^−5^ g. Thereafter, the gelatin is blended with a known volume of distilled water to obtain the targeted weight fraction gel composition (25% wt). Furthermore, the gelatin solution was agitated using an Ultrasonic Bath (J. P. Selecta.S.A., 120 W) for 30 min to dissolve the fish collagen adequately and thus, forming a homogenous gel.

The weight of the obtained gelatin was measured in a Mettler AE-240 balance with an accuracy of 5 10^–5^ g. Afterwards, The weighted gelatin was blended in the selected volume of deionized water. Ionogels, with percent weight concentrations of 25% wt for the gelatin. An ultrasonic bath (J. P. Selecta.S.A., 120 W) was used to obtain the correct dispersion. The FIL employed 1-ethyl-3-methylpyridinium perfluorobutanesulfonate ([C_2_C_1_py][C_4_F_9_SO_3_]) (>99% mass fraction purity) was obtained by Iolitec (Heilbronn, Germany). [C_2_C_1_py] [C_4_F_9_SO_3_] features at 30 °C a density of 1.52 g·cm^−3^ with a viscosity of 150.3 mPa s, and molar volume of 279.01 cm^3^·mol^−1^.

For the therapeutic agents, (MTM) was purchased from EntreChem SL and Doxorubicin (DOX) was purchased from MedChemExpress.

### 4.2. Emulsions and Ionogels Preparation

The selected Ionogels concentrations (shown in Table 2) were obtained at different ionic liquid/water ratio (1, 5, 10, 15, 25% wt of FIL), maintaining a fixed weight percentage of 25% wt gelatin of the total formulation. The afforded Ionogels were mixed with a sonicator to obtain a homogeneous blend for 30 min at 50 °C with a 5 L Selecta low-power ultrasonic bath (J.P. Selecta S.A., Spain) with an ultrasonic frequency of 40 kHz and output power of 120 W, as was proved to remove air bubbles as the gelatin dissolution is obtained above 35 °C (due to the interruption of physical cross-linking).

The IL/Water emulsions were formed at the same ionic liquid/water concentrations as their corresponding Ionogels. Emulsions were vigorously mixed by a vortex (LBX V05 series, Labbox Labware S.L., Barcelona, Spain) for good fluidity and stability.

### 4.3. Preparation of Drug-Loaded Gelatin Hydrogel

Drug-loaded gelatin solutions were prepared by passive loading methodology. Gelatin solutions were heated for 30 min at 55 °C. After that, 1 mg of the drug was added to the gelatin hydrogel to reach a final concentration of 1 mg/mL, forming thus Ionogels with the therapeutic agent.

### 4.4. Methods

**Zeta Potential and Dynamic Light Scattering**. The Zeta potential (ζ) and hydrodynamic sizes of IL and IL/Water samples were determined by a Zetasizer Nano S (Malvern Instruments, Malvern, UK) equipped with a 4 mV He–Ne laser operating at a wavelength of 633 nm. Experiments were conducted at 25 °C, in disposable plastic cuvettes and using a non-invasive back-scattering angle of 173°. Prior to the measurements, all aqueous solutions were mildly shaken with a vortex system and filtered using sterile Millex PVDF filters with a pore diameter of 0.822 μm. Results are based on at least 3 parallel runs with 20 acquisitions each.

**Density Measurements.** The densities, ρ, were measured in the thermal range between 10 to 40 °C with a vibrating U-tube densimeter (DMA 4500, Anton Paar, Graz, Austria) and uncertainty of 5·10^−4^ g·cm^−3^ [86]. The measurements were carried out 3 times and the obtained values were given as the mean with a maximum relative standard deviation of 0.5%.

**Raman Spectroscopy.** Raman measurements were carried out with a micro-Raman spectrometer LabRAM HR from Horiba Jobin with a laser at 532 nm for the IL/Water emulsions and 785 nm for both DOX loaded IL/Water emulsions and the Ionogels. The spectral resolution was around 1 cm^−3^ and a 10× microscope objective was used. The Raman spectra were studied by multivariate analysis using Eigenvectors developed by Horiba, the Raman spectra were pre-processed by SNV in order to normalize the spectra in the spectral region, to correct the noise remaining from the basic pre-processing done in the Raman spectrometer and its iCRaman software. The algorithm used for the input model is a Singular Value Decomposition (SVD) with full validation of the results.

**Fourier-Transform Infrared Spectroscopy by Attenuated Total Reflectance** (ATR-FTIR) was conducted using a Spectrometer Nicolet 6700, fitted with a source IR -Turbo fitted with a detector DTGS. The spectra of the Ionogels were obtained in a beamsplitter of KBr. The number of background scans was set to 34 and the tests were performed at ambient temperature with a spectral resolution of 4 cm^−1^. The Ionogels were deposited in the gold holder fitted with a humid chamber to prevent evaporation during the measurement. Background readings were subtracted from sample readings.

**Rheological Experiments.** The mechanical behavior was investigated using a Physica MCR 101 rheometer (Anton Paar, Graz, Austria) and a rotational Discovery Hybrid Rheometer DHR-2 (TA Instruments, New Castle, DE, USA) capable of controlling torques between 0.5 μN·m and 125 mN·m the former and between 2 nN·m and 200 mN·m for the latter. The viscosity values of the pure ionic liquid and the IL/Water obtained in this work from rotational and oscillatory tests present deviations lower than 8%.

The IL/Water emulsions flow tests were conducted with the Physica MCR 101 device at shear rates from 0.1 s^−1^ to 100 s^−1^ using a concentric cylinder geometry (cup: CC27/T200/SS with an inner diameter of 28.9 mm; and bob: B-CC27/P6 bob with outer diameter of 26.7 mm). The temperature was controlled by a C-PTD200 cylinder Peltier system. The declared uncertainty of the shear viscosity results obtained was below 4%. Oscillatory strain sweeps were also conducted for the IL/Water emulsions with the DHR-2 rheometer. A coaxial cylinder geometry together with a Peltier jacket was selected for the experiments. The geometry consisted of an external cup (diameter: 30.4 mm) and a bob (diameter: 28.0 mm, operating gap: 1.2 mm) in which the frequency was fixed at 0.05 Hz and the strain was increased from 1 to 1000%.

The Ionogels oscillatory analyses were conducted at 10 °C with the Physica MCR 101 rheometer operating with a plate-plate geometry (PP 25/S) and a gap of 0.1 mm. The temperature was controlled by a Peltier P-PTD 200, which was placed on the bottom plate. First, to identify the linear viscoelastic range (LVR), strain sweeps were performed at a constant pulsation of 10 rad/s and strains ranging from 0.01 to 1000%. Then, the storage modulus G’ and the loss modulus G” were determined over the linear strain range using a constant strain of 0.1% and covering frequencies from 0.1 to 200 rad/s. In addition, a temperature sweep of an oscillatory experiment was also carried out, in a temperature range of 10 to 40 °C at a constant frequency of 1Hz and a strain of 0.1 to 1%.

Prior to experiments, solutions were preheated at 60 °C for 30 min to homogenize samples and ensure proper geometry filling, as well as to avoid air bubbles. All rheological measurements were performed in triplicate to report reproducible data.

The *syringe test* measurements were carried out with a geometry, PP25/S plate and a gap of 0.1 mm. The measurements involve 3 different stages [42] in which an steady strain of 0.1% at 22 °C is first applied. Afterwards, the temperature is raised to 37 °C at a steady shear rate of 100 s^−1^. The final step is to apply a strain of 0.1% at 37 °C. The reported data are given with deviations in the (complex) viscosities between repetitions less than 3%.

**Cryo-Scanning Electron Microscopy (Cryo-SEM).** The microstructures of the shark gelatin hydrogel (GE) and Ionogels at 5, 15, and 25% wt were probed in cryo mode. The samples were deposited on a gold 13 mm and frozen at −200 °C by means of liquid nitrogen; they were then immediately transferred under liquid nitrogen in a cryogen box, to the Baltec equipment (MODEL, MED-020). Afterwards, they were cut on the surface (with a thickness of 2 to 3 mm). The snapped samples were observed, and images were acquired with a scanning electron microscope (JEOL JSM-6700) at an acceleration voltage of 5 kV.

The morphology of the collapsed gelatin-based Ionogels after etching (where etching involves semi-drying) is then visualized which provides a coarse structure of the hydrogel [87]. Moreover, a quantitative morphological analysis was performed with image J. The Interval Plot graph exhibits the average equivalent diameter or Aspect Ratio for the porous structure, the error bars represent 95% confidence intervals.

**Atomic Force Microscope (AFM).** The surface morphology of the GE and Ionogels was also investigated to proved further complementary structural information to the cryo-SEM analyses. For Atomic Force Microscopy, the instrument used is Veeco’s Multimode 8 Nanoscope V, Tapping Mode measurement mode. The Cantilever used was NCHV-A (Tip Roc < 10 nm, Cantilever Antimony (n) Doped Si, K = 40 N/m, Frequency= 339–388 KHz). The images shown have been obtained using the 11.9 μm scanner. Processing Software: Nanoscope v8.10, Nanoscope Analysis, Gwyddion 2.3. For the preparation of the measurements, an aliquot is taken and deposited on a silicon substrate previously fixed with adhesive on a steel support. Measurements have been made in two different areas, taking images with different fields of view/magnifications (10 × 10, 5 × 5, 1.5 × 1.5 μm^2^ and 600 × 600 nm^2^). The quantitative determination of the three-dimensional roughness parameters (Sa, Sq, Sz, Ssk and Sku) was achieved after proceeding with a “Cylinder and Tilt” correction to erase plane inclination and thus, produce micrographs with insignificant dependence on geometric deformations. The morphological analysis was conducted without the interpolation in the pixels with missing areas, although, the images were depicted with the interpolation “Data Restore” to enhance the micrograph quality.

**Differential Scanning Calorimetry (DSC).** The thermal properties of dry shark gelatin, the ionic liquid, the IL/Water emulsions as well as the GE and Ionogels were analysed by differential scanning calorimetry using a Q2000 (TA Instruments, New Castle, DE, USA). The cold source consists of a mechanical cooling unit capable of reaching cell temperatures down to −90 °C. DSC calibration was performed according to manufacturer specifications and temperature and enthalpy corrections were conducted using indium as standard (melting point, T_m_ = 156.61 °C). The measuring chamber was purged with a continuous dry-nitrogen flow rate of 50 mL/min. Prior to the tests, GE and Ionogels were sonicated at 60 °C in a low-power ultrasound bath for half an hour to ensure the homogeneity of the gelatin solutions. Approximately 15 mg of sample were hermetically sealed in Tzero aluminium capsules able to withstand pressures up to 0.3 MPa. The following three-step thermal protocol was used for all the materials. Samples were first cooled down to 1 °C and maintained at those conditions for 5 min to ensure a uniform initial temperature within the studied materials. Then, three heating and cooling runs in the temperature range between 1 and 80 °C with a scanning rate of 5 °C/min were programmed. Finally, samples were subject to cooling and heating ramps between −80 and 80 °C at 1 °C/min. In the case of the dry gelatin, the last heating scan was extended up to 200 °C. The weights of the DSC pans were obtained before and after the experiments to prove the absence of mass loss occurs. The experimental uncertainties in enthalpy and temperature determinations are estimated as 1.2 J/g and 0.3 K, respectively.

**Modulated Differential Scanning Calorimetry (MDSC).** IL/Water emulsions were also analysed by means of Modulated Differential Scanning Calorimeter (MDSC) in the temperature range between −80 and 80 °C. The scanning rate was 1 °C/min while the modulation period and amplitude were selected to 60 s and 0.159 °C, respectively, to ensure heat-iso conditions (i.e., in the heating process the temperature never decreases during the modulation).

**SDS-PAGE (Sodium Dodecyl Sulfate Polyacrylamide Gel Electrophoresis).** Samples were analysed using SDS-PAGE according to Laemmli [88]. Briefly, gelatin samples were dissolved at 3 mg/mL in mixtures of ionic liquid: water with weight fractions from 0 to 33.3% of total solution weight. Each solution was diluted 1:2 in sample buffer containing 10.52% glycerol, 21% sodium dodecyl sulfate (SDS) (10%), 0.63% dithiothreitol (DTT), and 0.5-M Tris-HCl (pH 6.8) and heated for 5 min at 100 °C. Collagen from blue sharks extracted as previously described [89] was also dissolved at 1 mg/mL and treated similarly. A 15 μL aliquot of each sample was applied to wells in a 7% SDS polyacrylamide separating gel (100 mm × 750 mm × 0.75 mm) and electrophoresis was performed at 15 mA by a Mini-Protean II cell (Bio-Rad, Hercules, CA, USA). Thereafter, 0.04% Coomassie Blue in 25% ethanol (*v*/*v*) and 8% acetic acid (v/v) was applied for 2 h to stain the gels. Repeated washes of destaining solution (25% ethanol (*v*/*v*), 8% acetic acid (*v*/*v*)) were performed to eliminate the surplus of stain. The assessment of the molecular weight was conducted by protein standards from Thermo-Scientific (Waltham, MA, USA) Pageruler Unstained High Range (250–60 kDa) and Pageruler Plus Prestained Protein Ladder (250–10 kDa).

**SAXS-Small-Angle X-Ray Scattering WAXS-Wide Angle X-Ray Scattering.** The nanostructure of both IL-water emulsions, as well as their corresponding gelatin hydrogels, was investigated by X-ray scattering experiments at different length scales by simultaneous SAXS-WAXS measurements. In addition, the Il-Water emulsions and the gelatin IL-hydrogels were subjected to thermal treatment by using a Linkam HFSX350 capillary stage to monitor the structural evolution during the thermal transitions.

Small-angle X-ray scattering (SAXS) and wide-angle X-ray scattering (WAXS) experiments were conducted in NCD-SWEET beamline at ALBA synchrotron (Cerdanyola del Vallès, Spain) as well as at DUBBLE at ESRF. The energy of the X rays was set to 12 keV which is equivalent to a wavelength of 1 Å; and a beam size of ca. 100 μm × 100 μm (FWHM) for Alba experiments and ca. 300 μm × 300 μm for DUBBLE measurements. The SAXS patterns were acquired using a Pilatus 1 M detector that is characterized by an active surface of 981 × 1043 pixels of 172 μm × 172 μm at a sample to detector distance of ca 2.5 m whereas the WAXS patterns were collected using either a Rayonix LX255-HS or a Pilatus 300 K-W that possess a sensitive area of either 85 × 255 mm^2^ or 1472 × 195 pixels respectively and a corresponding pixel size of 40 μm× 40 μm or 172 μm × 172 μm. The scattering patterns of the SAXS (Silver behenate, AgBe) and WAXS (α-alumina, α-Al_2_O_3_) have been used to calibrate the scattering vector q, where q = 4πsinθ/λ and θ being half of the scattering angle and λ the beam wavelength. The collected 2D patterns were integrated using the Bubble [90] free software applying the required corrections such as the background subtraction amended by X-ray transmission through the sample as well as the normalization upon the incident beam intensity to avoid beam fluctuations. The intensity of the scattering vector was calibrated using the scattering of water as a calibrant.

The IL-water emulsions and the gelatin hydrogel were inserted in a glass capillary of 1.5 mm (diameter) and closed prior to being introduced in the Linkam heating stage. The samples have been subjected to a thermal protocol involving a cooling step from 22 °C to −40 °C and the subsequent heating step to 40 °C at a rate of 5 °C/min.

The IL/Water emulsions experimental reduced data were adjusted to the weakly correlated nanoscale mass fractal aggregates model by an in-house program [91,92] that use the minimization routine of MINUIT [93,94] program created in CERN in which I(q) is adjusted to:

(1) $$I\left(q\right)=\left\{\begin{array}{c} \frac{G}{1+kF_{0}\left(q\xi \right)}\exp\left(\frac{q^{2}R_{g}^{2}}{3}\right),\;\;q<q_{1}\\ \frac{D}{1+kF_{0}\left(q\xi \right)}q^{-D_{m}},\;\;q\geq q_{1} \end{array}\right.$$

The parameters to be fitted are *k*, *ξ*, *R_g_*, *G* and *D_m_*, where *k*, with 0 < *k* < 6 depict the degree of correlations over a distance *ξ*, *ξ* is the correlation distance, *G* is the exponential (Guinier) prefactor, *R_g_* is the radius of gyration, with 0 < *D_m_* < 3 as the fractal dimension of the aggregate.

The Ionogels integrated SAXS profiles were fitted to a heterogeneous sphere with a fractal structure which is described with a radius of gyration 𝑅𝑔 associated with the fractal size and the fractal dimension *D* that defines their compactness [11,95] with the previous program, and thus, the SAXS profile intensity (*I*(*q*)) was adjusted to:

(2) $$(\text{𝑞})=\frac{\left(D-1\right)\;\sin\left(\left(D-1\right)\;tan-1\left(q,\xi \right)\right)}{\left(D-1\right)q\xi \;{\left(1+q2\xi 2\right)}^{\frac{\left(D-1\right)}{2}}}$$

with 𝜉^2^ =$\frac{2Rg^{2}}{D\left(D+1\right)}$, 𝜉 being the correlation length.

**Solubility Test.** The solubility of DOX was determined visually, by adding DOX to three different combinations of ionic fluid (FIL) and H_2_O mixtures. The ratios of FIL/ H_2_O were 0.1 g/0.65 g, and 0.25 g/0.5 g. Excess amounts of drugs were added to the mixtures to reach saturation concentration.

Firstly, 1 mg of DOX was added to each mixture and mixed at 3000 rpm with a Fisherbrand™ Vortex for 5 min followed by 5 min of an ultrasonic bath at room temperature (22 °C); these steps were repeated to reach concentrations of 2, 5, and finally 10 mg/mL of DOX.

In another experiment, the solubility of DOX was also tested in FIL without water. Excess amounts of drug were added to the mixtures to reach saturation concentration, but only Vortex mixing was needed this time.

**Drug Release Experiments.** 200 μL of each drug-loaded gelatin were located in the dialysis membrane (molecular weight cut-off: 12–14 kDa) and incubated in 8 mL of phosphate-buffered saline (PBS, pH 7.4) at 37 °C with continued mixing with a stirrer (300 rpm) in an IKA Plate (RCT digital). At different intervals of incubation, 1 mL of releasing medium was removed and new PBS was added to maintain the volume constant. The drug release profile was examined by recording UV-Vis spectra for 8 days. The drug-release concentration was measured in a JASCO V650 UV-Vis spectrophotometer at 403 and 483 nm for MTM and DOX, respectively, using PBS as a reference. The drugs released were tested in duplicates.

## Figures and Tables

**Table 1:** The acronym that describes the generated Il/water emulsions under study with 1-ethyl-3-methylpyridinium perfluorobutanesulfonate named as IL.

Acronym of IL/Water	Sample Description (Mass Rate of IL/Water)
1IL/Water	1%wt IL:74%wt Water
5IL/Water	5%wt IL:70%wt Water
10IL/Water	10%wt IL:65%wt Water
15IL/Water	15%wt IL:60%wt Water
25IL/Water	25%wt IL:50%wt Water

**Table 2:** The acronym designates the Ionogels formed from the gelatin at a fixed weight ratio dispersed in the emulsions of IL in water at the corresponding concentrations under study.

Acronym of Ionogels	Sample Description (Mass Compositions of IonoGels) in % wt Water
GE	25%wt GE
IL	100% IL
GE/1IL	25%wt GE + 1%wt IL
GE/5IL	25%wt GE + 5%wt IL
GE/10IL	25%wt GE + 10%wt IL
GE/15IL	25%wt GE + 15%wt IL
GE/25IL	25%wt GE + 25%wt IL

**Table 3:** Zeta Potential and Size dimension with the standard deviations for the single IL and its IL/Water emulsions.

Samples	Average Size (nm) Mean Peak	Zeta Potential (mV)
IL	NA	−128 ± 19.7
1IL/Water	4.0 ± 0.4	−12.8 ± 2.4
5IL/Water	4.0 ± 0.2	−12.4 ± 3.4
10IL/Water	4.1 ± 0.0	−12.5 ± 3.0
15IL/Water	4.7 ± 0.0	−12.0 ± 2.3
25IL/Water	6.0 ± 0.1	−11.3 ± 0.9

**Table 4:** Accumulative enthalpies are associated with either endothermic or exothermic transitions of IL/Water mixtures in the temperature range between −80 and −15 °C.

Samples	Exothermic Transitions	Endothermic Transitions
	ΔH (J/g)	ΔH (J/g)
1IL/Water	0.56	0.58
5IL/Water	2.03	2.08
10IL/Water	3.96	3.94
15IL/Water	4.63	4.70
25IL/Water	11.74	12.12

**Table 5:** Values were obtained from TGA and DTGA analyses of Dry GE, GE, GE/15IL and IL. Onset temperatures, Tonset; maximum temperatures, Tmax; and weight loss (%) at °C/min.

Samples	Steps	T_Onset_ (°C)	T_Max_ (°C)	Weight Loss (%)
Dry GE	1	39.75	89.04	−12.44
	2	247.76	311.24	−52.99
	3	560.70	641.59	−23.62
IL	1	411.95	437.33	−94.42
GE	1	54.69	100.48	−76.29
	2	250.65	310.53	−15.98
GE/15IL	1	83.68	100.14	−66.84
	2	255.35	312.11	−19.09
	3	427.70	479.13	−11.43

**Table 6:** Assignments of IR bands to the secondary structure of GE and Ionogels.

Samples	Wavenumber (cm^−1^)				
	β-Sheet	Random Coil	Triple α-Helix	3_10_-Helix	β-Turn
GE	1628, 1633	1641	1649, 1660	1670	1680, 1691
GE/1IL	1628, 1634	1643	1649, 1660	1670	1680, 1691
GE/5IL	1628, 1635	1643	1649, 1660	1670	1680, 1691
GE/10IL	1628, 1637	-	1649, 1660	1672	1682, 1691
GE/15IL	1628, 1637	-	1649, 1660	1671	1681, 1691
GE/25IL	1628, 1637	-	1649, 1660	1672	1682, 1691

**Table 7:** Temperature and enthalpy associated with gelatin unfolding *.

Samples	Heating		Cooling
	T (ºC)	ΔH (J/g)	T (ºC)
GE	23.2	4.2	7.8
GE/1IL	21.7	3.7	7.1
GE/5IL	21.4	3.2	7.1
GE/10IL	20.1	3.0	5.7
GE/15IL	20.5	2.8	5.5
GE/25IL	20.8	2.6	7.0

* Values obtained from DSC scans at 5 °C/min. Reported temperatures correspond to peak value (maximum deviation of DSC heat flow signal from baseline).

**Table 8:** 2D Roughness parameters were calculated from the 10 × 10 μm^2^ field of view images obtained by AFM of zone 1 and 2.

AFM/2D Amplitude Roughness Parameters/Imaging 10 × 10 μm^2^					
	Ra (nm)	Rq (nm)	Rz (nm)	Skewness (Ssk)	Kurtosis (Sku)
Zone 1	3.54	5.00	49.80	1.87	11.30
Zone 2	2.38	3.09	33.40	0.62	4.07

**Table 9:** Roughness parameters were calculated from the 10 × 10 μm^2^ field of view images obtained by AFM of zone 1 and 2 of GE/5IL and GE/25IL.

AFM/Parameters Amplitude 3D Roughness/Imágenes 10 × 10 μm^2^						
		Sa (nm)	Sq (nm)	Sz (nm)	Skewness (Ssk)	Kurtosis (Sku)
GE/25IL	Zone 1	9.15	19.0	316	−5.35	43.1
	Zone 2	8.32	14.6	209	−3.97	27.9
GE/5IL	Zone 1	3.77	4.54	42.3	−0.091	2.87
	Zone 2	1.43	1.89	32.0	0.889	6.43

## Supplementary Materials

The following are available online at https://www.mdpi.com/article/10.3390/gels8090594/s1, Table S1: pHs of the IL and IL/H_2_O emulsions; Table S2: Density, *ρ* (g·cm^−3^), as Function of Ionic Liquid Mass Fraction, w_IL_, for the emulsions (IL/Water) at 10, 20, 25, 30 and 40 °C; Table S3: Summary of the fitting of the IL/Water emulsions SAXS profiles to adjusted to the weakly correlated nanoscale mass fractal aggregates model with a radius of gyration 𝑅𝑔 associated with the fractal size and the fractal dimension D that define their compactness and Ionogels and 𝜉 being the correlation length. The 1IL/Water was not fitted due to the lack of structure and therof the fitting parameters are filled in the table as not applicable (NA); Figure S1: DSC thermogram of the cooling and heating cycles obtained for [C_2_C_1_py][C_4_F_9_SO_3_] ionic liquid at 1 °C/min and 5 °C/min; Figure S2: DSC thermogram (a,b) Cooling ramp of (—) Water, (—) 1IL/Water, (—) 5IL/Water, (—) 10IL/Water, (—) 15IL/Water, (—) 25IL/Water and (—) IL at 1 °C/min; (c) Cooling ramp of (—) Water at 1 °C/min; (d) Heating ramp of (—) Water at 1 °C/min; Figure S3: (a) Total; (b) reversing and; (c) non-reversing heat fluxes from heating MDSC runs at 1 °C/min for some representative IL/water emulsions: (—) 1IL/Water, (—) 5IL/Water, and (—) 25IL/Water; Figure S4: Raman spectra of (—) 5IL/Water, (—) 10IL/Water, (—) 15IL/Water, (—) 25IL/Water; Table S3: Summary of the fitting of the 25IL/Water emulsion loaded with DOX SAXS profiles to adjusted to the weakly correlated nanoscale mass fractal aggregates model with a radius of gyration 𝑅𝑔 associated with the fractal size and the fractal dimension D that define their compactness and Ionogels and 𝜉 being the correlation length; Figure S5: Raman spectra of: (a) 25IL/Water loaded with DOX; (b) and the corresponding PCA; Table S4: Main FTIR vibration bands associated with IL and gelatin nanostructure; Figure S6: FTIR Spectra of Amide I and Amide II for: (—) GE, (—) GE/1IL, (—) GE/5IL, (—) GE/10IL, (—) GE/15IL and (—) GE/25IL; Table S5: FTIR bands of Amide I and Amide II for: GE, GE/1IL, GE/5IL, GE/10IL, GE/15IL and GE/25IL; Figure S7: (a) Raman spectra of the Ionogels and; (b) their corresponding PC1 an PC2; Figure S8: DSC cooling and heating thermograms obtained for dry shark gelatin; Table S6: Enthalpies and Temperature of crystallization of water in the different hydrogel and Ionogels; Table S7: Summary of the fitting of the SAXS profiles to a a heterogeneous sphere with fractal structure with with a radius of gyration 𝑅𝑔 associated with the fractal size and the fractal dimension D that define their compactness and Ionogels and 𝜉 being the correlation length; Figure S9: Cryo-scanning electron microscopy of: (a,b) Shark gelatin hydrogel, (a) X2000; (b) X37000; (c) GE/5IL at X1600; (d) GE/15IL at X20000; (e) and (f) GE/25IL at X15000; Figure S10: Cryo-scanning electron microscopy at X5000 of: (a) Shark gelatin hydrogel; (b) GE/5IL; (c) GE/15IL; (d) GE/25IL; Figure S11: Statistical analysis of cell size-distributions and cell aspect-ratio distributions obtained from the three micrographs captured at X5000 magnification. (a) Interval plot of pore size, (b) Interval plot of aspect ratio, (c) Histogram of pore size, (d) Historgram of aspect ratio The The error bars represent 95% confidence intervals; Figure S12: AFM images of shark gelatin hydrogel. (a) Zone 1 at a magnification of 10 × 10 μm^2^, (b) Zone 2 at a magnification of 10 × 10 μm^2^. (c) Image 2D with a field of view 1.6 × 1.6 μm^2^, (d) Image 2D with a field of view 849.5 × 849.5 μm^2^, (e) Image 2D in Zone 1 at a magnification of 5 × 5 μm^2^, (f) Zone 2 at a magnification of 5 × 5 μm^2^; (g), (h) Zone 1, Image 3D; Figure S13: Zoom images 3D with magnifications 120 × 120 nm^2^ of shark gelatin hydrogel. (a) Zone 1; (b) Zone 2; Figure S14: (a) Cryo-SEM images; (b) Image of Zone 1 at a magnification of 0.84 × 0.84 μm^2^; (c) Image of Zone 2 at a magnification of 1.1X1.1 μm^2^ of hydrogel gelatin; Figure S15: (a) Zone 2 of GE/25IL; (b) Zone 2 of GE/5IL Topographic 2D images Zone 2, 10 × 10 μm^2^; (c) Zone 1, Image 2D Field of View 1.5 × 1.5 μm^2^ of GE/25IL; (d) Zone 1, Image 3D Field of View 1.5 × 1.5 μm^2^ of GE/25IL; (e) Zone 1, Image 2D, Field of View 1.5 × 1.5 μm^2^ of GE/5IL; (f) Zone 1, Image 3D Field of View 1.5 × 1.5 μm^2^ of GE/25IL; (g) GE/5IL of Zone 1; (h) GE/5IL of Zone 2; Figure S16: Imaging of WLOP method. (a) 2D image at 5x_2x (443.2 × 582.5 μm^2^; (b) 20x_2x (110 × 144 μm^2^; (c) 250x_2x (44 × 58 μm^2^); (d) 3D magnification 5X_FOV 2X (443.2 × 582.5 μm^2^; (e) 3D magnification 20X_2X (110 × 144μm^2^), (f) 3D magnification 50X_2X (44 × 58 μm^2^); Table S8: Roughness parameter values obtained by WLOP of shark gelatin hydrogel; Figure S17: Complex viscosity variation (filled ◯); and storage modulus (G′, filled Δ) and loss modulus (G″, open Δ) versus temperature of gelatin hydrogel (orange color) and Ionogel (fuchsia color).
