# Supplementary material for: Ionogels Derived from Fluorinated Ionic Liquids to Enhance Aqueous Drug Solubility for Local Drug Administration
Source: Gels. 2022 Sep 16;8(9):594. doi: 10.3390/gels8090594 (PMC9498591; doi:10.3390/gels8090594)
Supplement: Supplementary file 1 [file gels-08-00594-s001.zip › gels-1845344-supplementary.pdf]

*Suplemmentary Materials*

# Ionogels Derived from Fluorinated Ionic Liquids to Enhance Aqueous Drug Solubility for Local Drug Administration

**Carolina Hermida-Merino [1], David Cabaleiro [1], Carlos Gracia-Fernández [2], Jesus Valcarcel [3], José Antonio Vázquez [3], Noelia Sanz [4], Martín Pérez-Rodríguez [1], Maria Arenas-Moreira [5], Dipanjan Banerjee [6,7], Alessandro Longo [8,9], Carmen Moya-Lopez [10], Luis Lugo [1], Patrice Bourson [10], Ana B. Pereiro [11], Georges Salloum-Abou-Jaoude [12], Iván Bravo [5], Manuel M. Piñeiro [1,*] and Daniel Hermida-Merino [1,6,*]**

**Table S1.** pHs of the IL and IL/$H_2O$ emulsions.

| Samples | pH | T (°C) |
|---|---|---|
| $H_2O$ | 6.23 | 27.4°C |
| 1IL/$H_2O$ | 7.44 | 28.5°C |
| 5IL/$H_2O$ | 7.45 | 28.6°C |
| 10IL/$H_2O$ | 7.49 | 29.1°C |
| 15IL/$H_2O$ | 7.51 | 29.1°C |
| 25IL/$H_2O$ | 7.51 | 29.1°C |
| IL | 6.67 | 27.9°C |

**Table S2.** Summary of the fitting of the IL/Water emulsions SAXS profiles to adjusted to the weakly correlated nanoscale mass fractal aggregates model with a radius of gyration $Rg$ associated with the fractal size and the fractal dimension D that define their compactness and Ionogels and $\xi$ being the correlation length. The 1IL/Water was not fitted due to the lack of structure and therof the fitting parameters are filled in the table as not applicable (NA).

| | 1IL/Water | 5IL/Water | 10IL/Water | 15IL/Water | 25IL/Water |
|---|---|---|---|---|---|
| $R_g$ | NA | 1.73 | 1.72 | 1.68 | 1.55 |
| $\xi$ | NA | 2.99 | 2.82 | 2.5 | 2.34 |
| $D_f$ | NA | 3.28 | 3.55 | 4 | 4 |

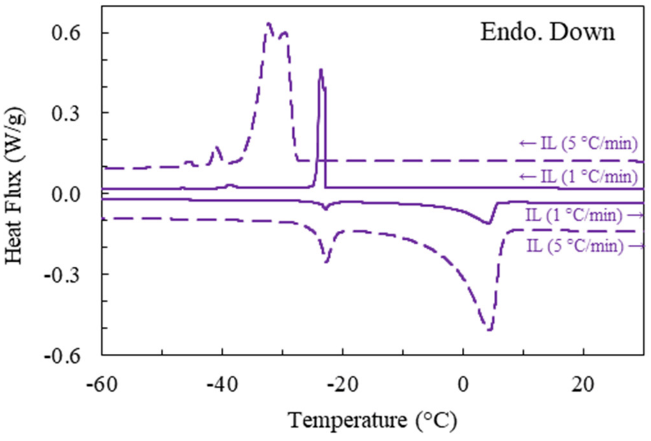


**Figure S1.** DSC thermogram of the cooling and heating cycles obtained for [$C_2C_1$py][$C_4F_9SO_3$] ionic liquid at 1 °C/min and 5 °C/min.

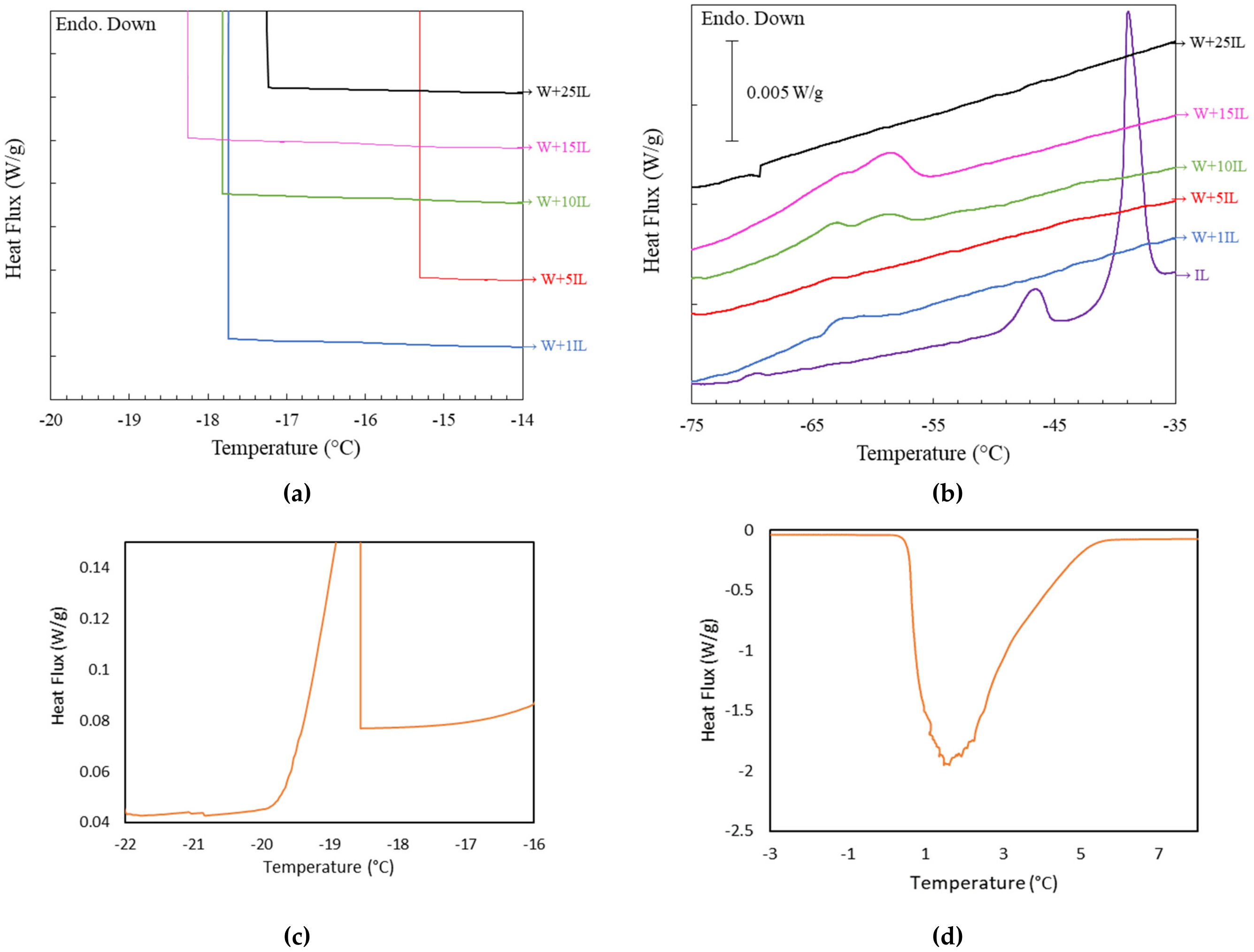


**Figure S2.** DSC thermogram (**a**, **b**) Cooling ramp of (—) Water, (—) 1IL/Water, (—) 5IL/Water, (—) 10IL/Water, (—) 15IL/Water, (—) 25IL/Water and (—) IL at 1 °C/min; (c) Cooling ramp of (—) Water at 1 °C/min; (d) Heating ramp of (—) Water at 1 °C/min.

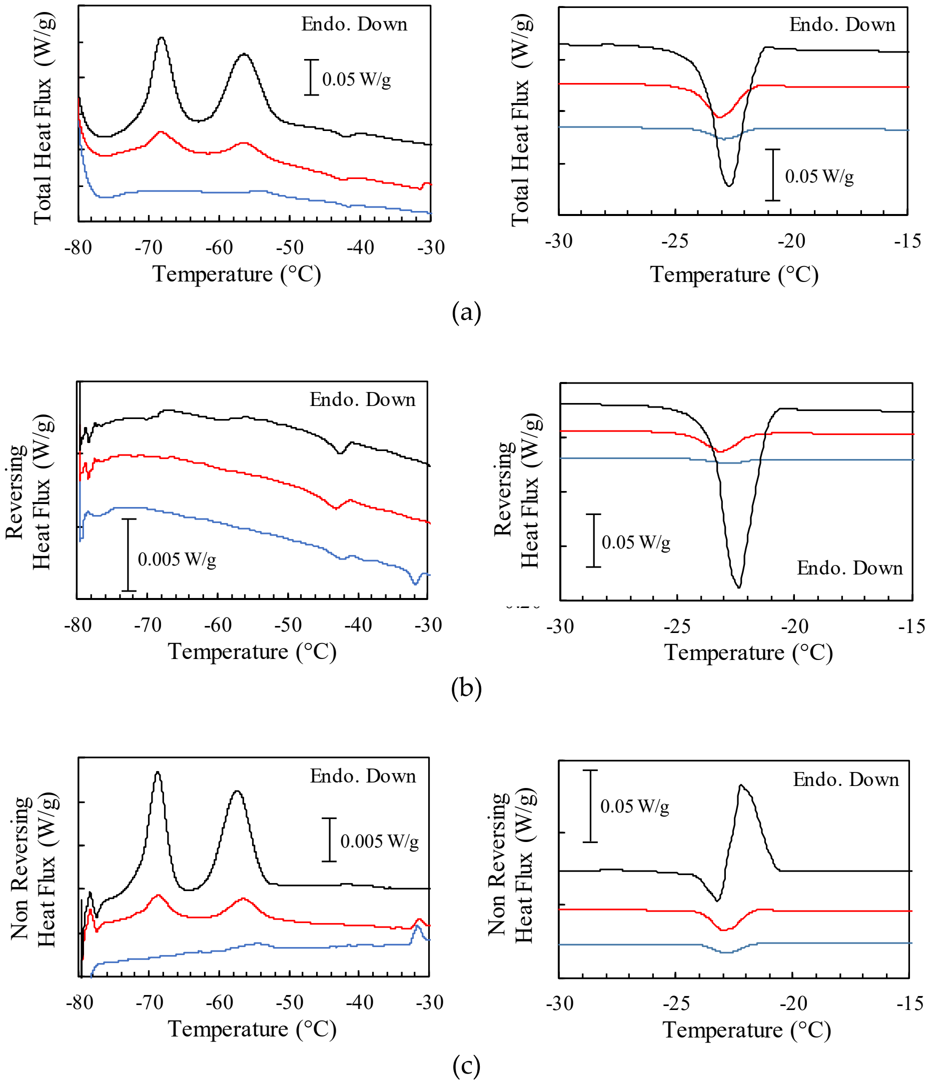


**Figure S3.** (**a**) Total; (**b**) reversing and; (**c**) non-reversing heat fluxes from heating MDSC runs at 1 °C/min for some representative IL/water emulsions: (—) 1IL/Water, (—) 5IL/Water, and (—) 25IL/Water.

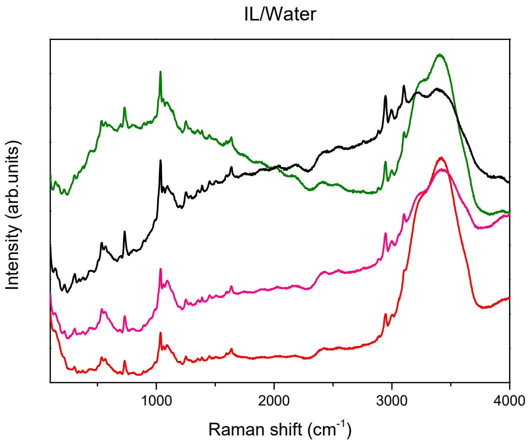


**Figure S4.** Raman spectra of (—) 5IL/Water, (—) 10IL/Water, (—) 15IL/Water, (—) 25IL/Water.

**Table S3.** Summary of the fitting of the 25IL/Water emulsion loaded with DOX SAXS profiles to adjusted to the weakly correlated nanoscale mass fractal aggregates model with a radius of gyration $Rg$ associated with the fractal size and the fractal dimension D that define their compactness and Ionogels and $\xi$ being the correlation length.

| | 25IL/Water/ DOX_1mg/ml | 25IL/Water/ DOX_2mg/ml | 25IL/Water/ DOX_5mg/ml | 25IL/Water/ DOX_10mg/ml |
|---|---|---|---|---|
| $R_g$ | 1.64 | 1.66 | 1.78 | 1.07 |
| $\xi$ | 2.31 | 2.3 | 2.36 | 1.63 |
| $D_f$ | 4.00 | 3.96 | 3.75 | 3.8 |

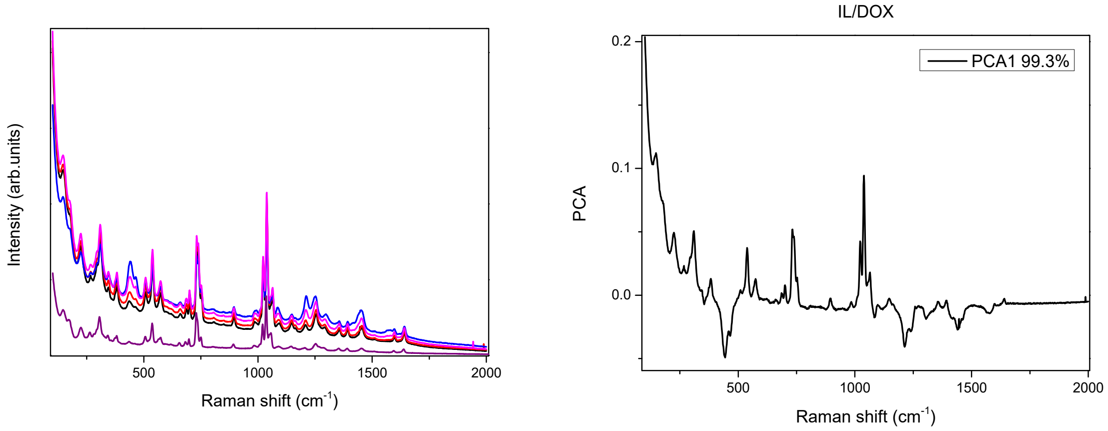


**(a)** **(b)**

**Figure S5.** Raman spectra of: (**a**) 25IL/Water loaded with DOX; (**b**) and the corresponding PCA.

**Table S4.** Main FTIR vibration bands associated with IL and gelatin nanostructure.

| Functional group | GE | GE/1IL | GE/5IL | GE/10IL | GE/15IL | GE/25IL | IL |
|---|---|---|---|---|---|---|---|
| | Wavenumber (cm$^{-1}$) | | | | | | |
| **N-H , O-H** | **3321** | **3334** | **3272** | **3353** | **3353, 3300** | 3353, 3300 | --- |
| **Aliphatic tension CH** | 2850-3000 | 2850-3000 | 2850-3000 | 2850-3000 | 2850-3000 | 2850-3000 | 2951, 3000 |
| **Aromatic CH tension** | --- | --- | --- | --- | --- | --- | 3067 |
| **Aliphatic CH flexion** | 1337, 1458 | 1388, 1458 | 1384, 1457 | 1381, 1457 | 1338, 1457 | 1338, 1457 | 1390, 1459 |
| **Amide I,II,III** | 1633, 1553, 1250 | 1634, 1553, 1250 | 1635, 1552, 1250 | 1637, 1552, 1251 | 1637, 1551, 1251 | 1637, 1550, 1251 | --- |
| **Tension C-O,C-O-C carbohydrates** | 1163, 1082 | 1163, 1082, 1064 | 1137,1082 , 1062 | --- | --- | --- | --- |
| **Water** | 600 | 600 | 600 | 600 | 600 | 600 | --- |
| **C-F ($CF_2$ group)** | --- | --- | 1130-1290 | --- | 1137-1290 | 1137-1290 | 1207-1253, 1131 |

| | | | | | | | |
|---|---|---|---|---|---|---|---|
| **C=C (pyridinium ring)** | --- | --- | --- | --- | --- | --- | 1638-1600 |
| **C=N (pyridinium ring)** | --- | --- | --- | --- | --- | --- | 1508, 1459 |
| **Pyridinium ring strain** | --- | --- | 1000 | 1000 | 1007 | 1000 | 1006 |
| **S-O stress mode ($SO_3$ group)** | --- | --- | 1020, 1060 | 1000, 1061 | 1000, 1060 | 1000, 1060 | 1055, 1048, 1020 |
| **S-O bending mode ($SO_3$ group)** | --- | --- | 600-700 | 600-700 | 600-700 | 600-700 | 600-700 |
| **S=O bond ($SO_3$ group)** | --- | --- | 1350 | 1352 | 1352 | 1352 | 1352 |
| **Flexion O=S=O** | --- | --- | --- | --- | 525 | 525 | 522-530 |

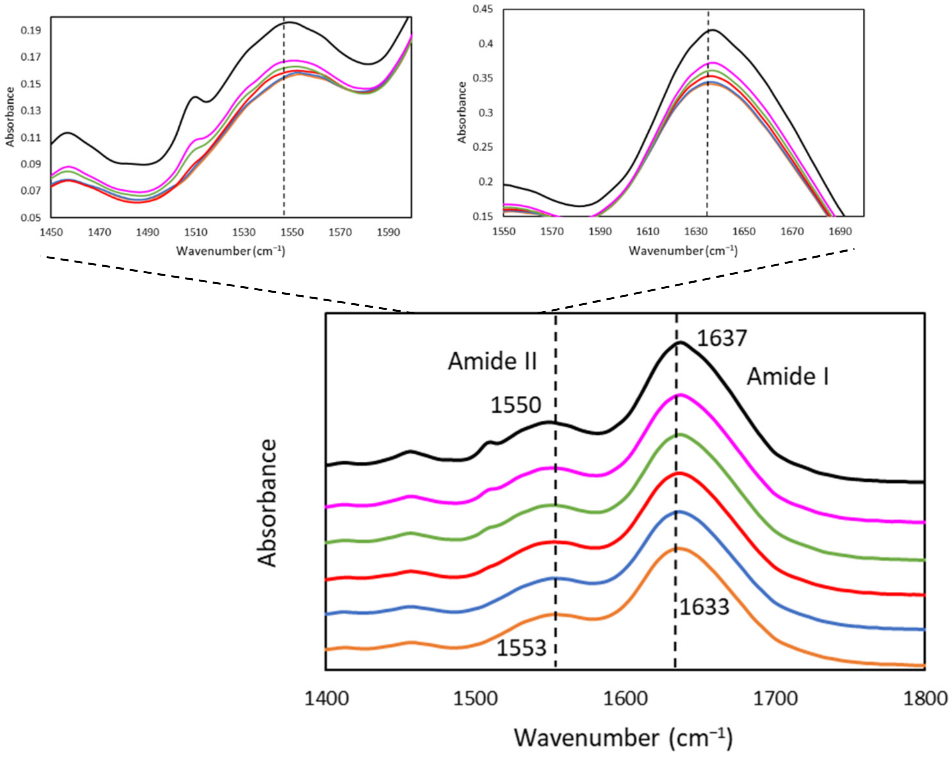


**Figure S6.** FTIR Spectra of Amide I and Amide II for: (—) GE, (—) GE/1IL, (—) GE/5IL, (—) GE/10IL, (—) GE/15IL and (—) GE/25IL.

**Table S5.** FTIR bands of Amide I and Amide II for: GE, GE/1IL, GE/5IL, GE/10IL, GE/15IL and GE/25IL.

| Samples | Amide I ($cm^{-1}$) | Amide II ($cm^{-1}$) |
|---|---|---|
| GE | 1633 | 1553 |
| GE/1IL | 1634 | 1553 |
| GE/5IL | 1635 | 1552 |
| GE/10IL | 1637 | 1552 |
| GE/15IL | 1637 | 1551 |
| GE/25IL | 1637 | 1550 |

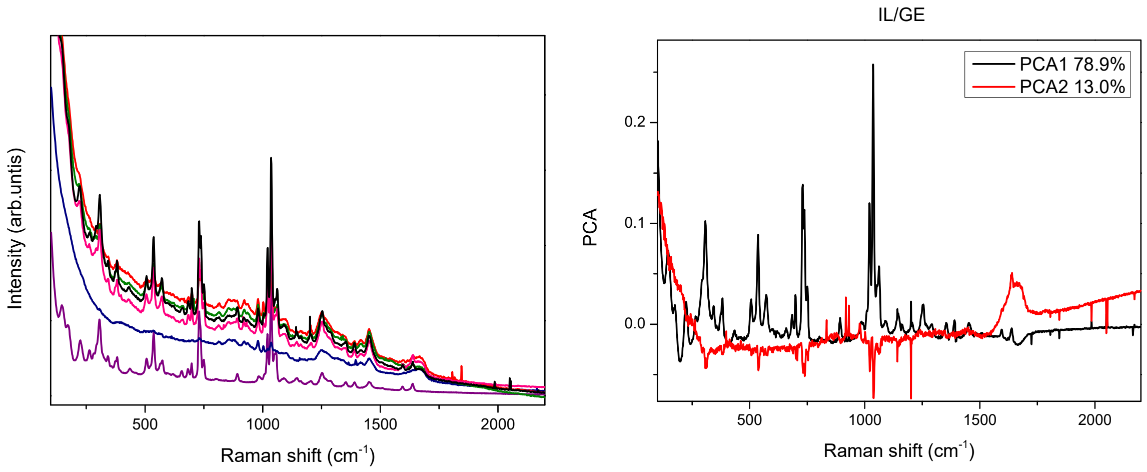


**(a)** **(b)**

**Figure S7.** (**a**) Raman spectra of the Ionogels and; (**b**) their corresponding PC1 an PC2.

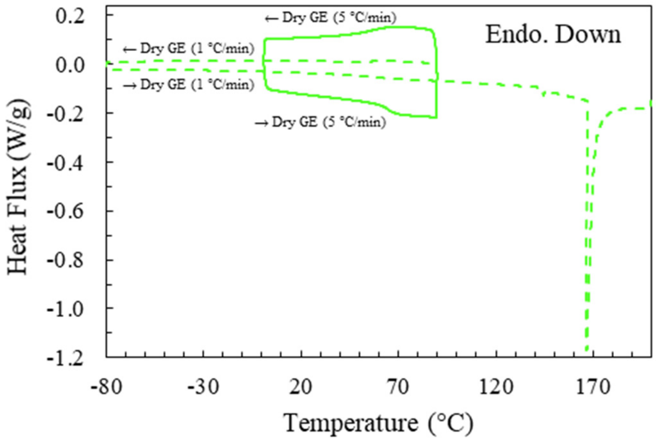


**Figure S8.** DSC cooling and heating thermograms obtained for dry shark gelatin.

**Table S6.** Enthalpies and Temperature of crystallization of water in the different hydrogel and Ionogels.

| | GE | IL | W | *T* (ºC) | *Δh* (J/g) |
|---|---|---|---|---|---|
| GE | 25 | 0 | 75 | -0.29 | 178.3 |
| GE+1IL | 25 | 1 | 74 | - 1.25 | 152.6 |
| GE+5IL | 25 | 5 | 70 | -1.09 | 149.3 |
| GE+10IL | 25 | 10 | 65 | -1.10 | 164.4 |
| GE+15IL | 25 | 15 | 60 | -1.43 | 135.6 |
| GE+25IL | 25 | 25 | 50 | -2.04 | 110.2 |
| Water | 0 | 0 | 100 | 1.47 | 308.7 |

**Table S7.** Summary of the fitting of the SAXS profiles to a a heterogeneous sphere with fractal structure with with a radius of gyration $Rg$ associated with the fractal size and the fractal dimension D that define their compactness and Ionogels and $\xi$ being the correlation length.

| | Ge/1IL 10°C | Ge/1IL 25°C | Ge/5IL 10°C | Ge/5IL 25°C | Ge/10IL 10°C | Ge/10IL 25°C | Ge/15IL 10°C | Ge/15IL 25°C | Ge/25IL 0°C | Ge/25IL 25°C |
|---|---|---|---|---|---|---|---|---|---|---|
| $R_g$ | 0.89 | 0.92 | 1.00 | 1.06 | 1.00 | 1.08 | 1,00 | 1.06 | 1.01 | 0.98 |
| $\xi$ | 1.22 | 2.13 | 1.68 | 2.46 | 1.99 | 1.92 | 2.13 | 1.62 | 1.28 | 1.14 |
| $D_f$ | 3.27 | 2.63 | 3.0 | 2.6 | 2.92 | 2.93 | 2.53 | 2.9 | 3.84 | 3.22 |

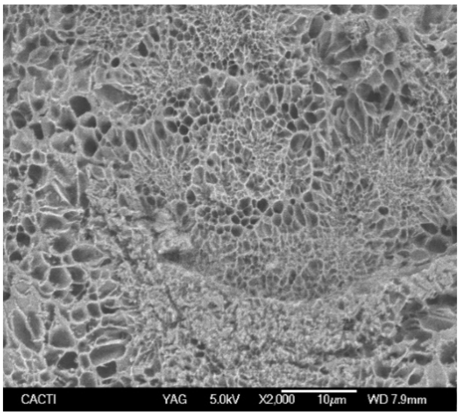

(**a**)

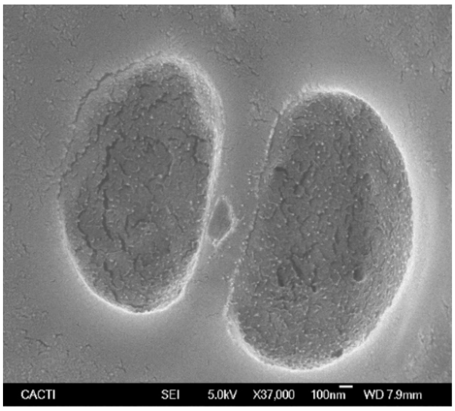

(**b**)

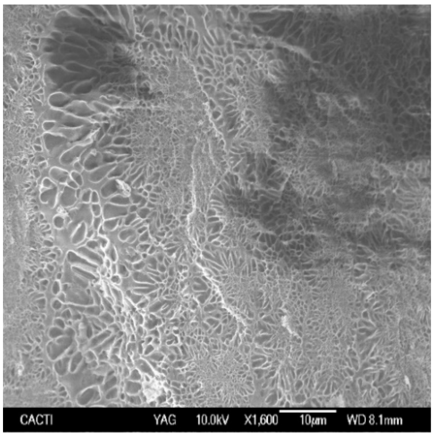

(**c**)

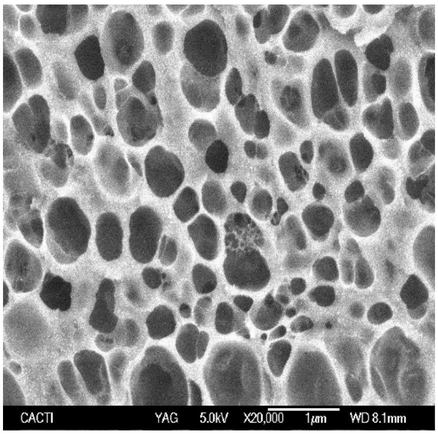

(**d**)

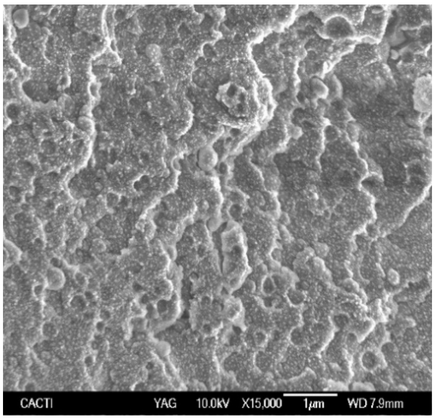


(**e**)

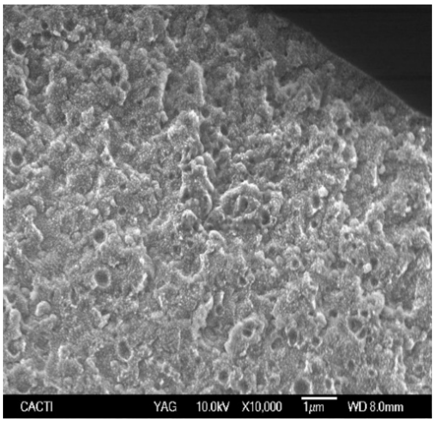


(**f**)

**Figure S9.** Cryo-scanning electron microscopy of: (**a**, **b**) Shark gelatin hydrogel, (**a**) X2000; (**b**) X37000; (**c**) GE/5IL at X1600; (**d**) GE/15IL at X20000; (**e**) and (**f**) GE/25IL at X15000.

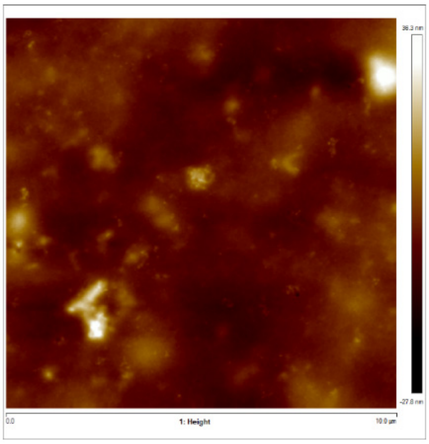

(a)

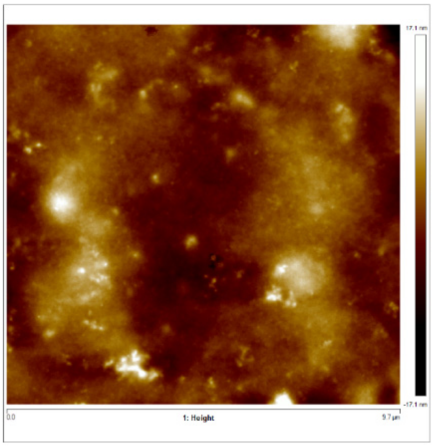

(b)

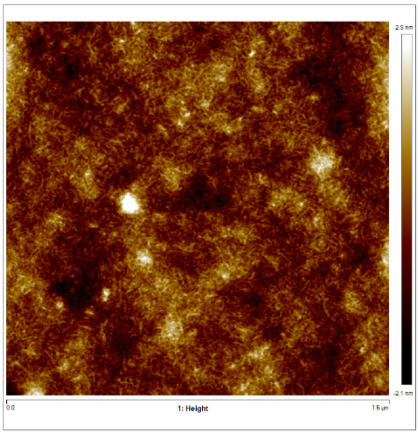

(c)

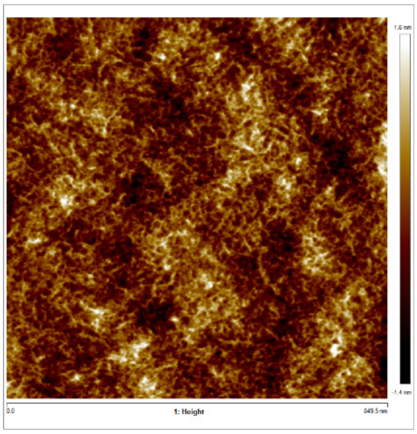

(d)

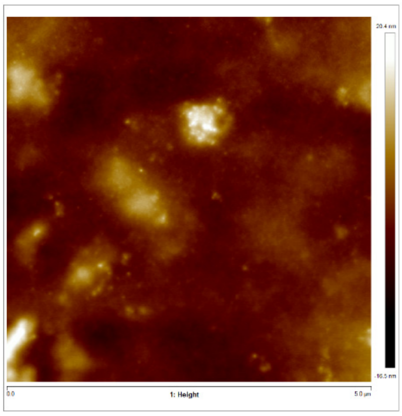


(e)

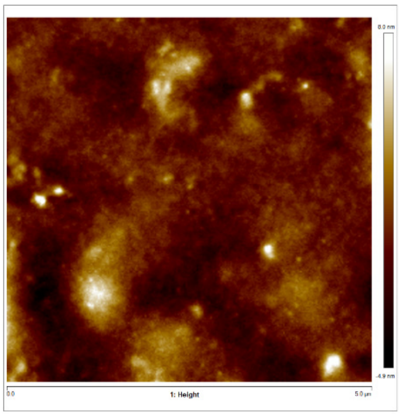


(f)

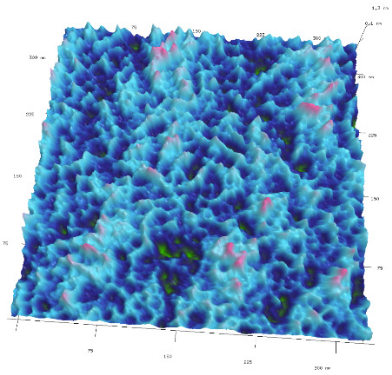

(g)

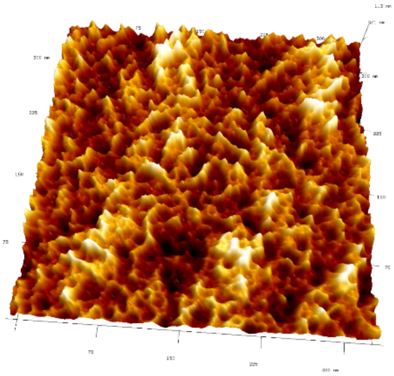

(h)

**Figure S10.** FM images of shark gelatin hydrogel. (**a**) Zone 1 at a magnification of 10x10 µm$^2$, (**b**) Zone 2 at a magnification of 10x10 µm$^2$. (**c**) Image 2D with a field of view 1.6X1.6 µm$^2$, (**d**) Image 2D with a field of view 849.5X849.5 µm$^2$, (**e**) Image 2D in Zone 1 at a magnification of 5x5 µm$^2$, (**f**) Zone 2 at a magnification of 5x5 µm$^2$; (**g**), (**h**) Zone 1, Image 3D.

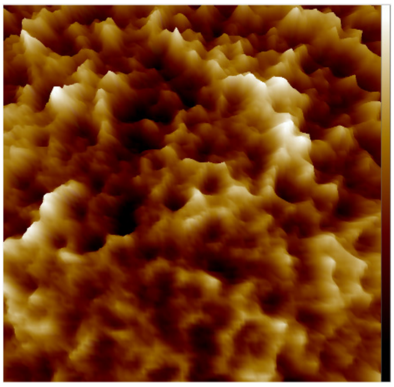

(**a**)

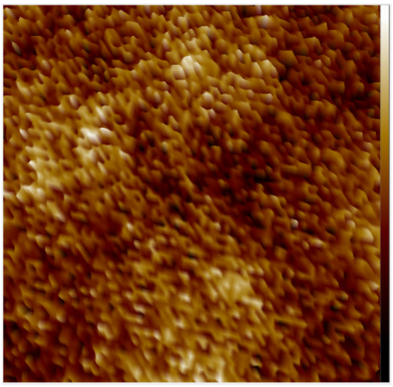

(**b**)

**Figure S11.** Zoom images 3D with magnifications 120x120 nm$^2$ of shark gelatin hydrogel. (**a**) Zone 1; (**b**) Zone 2.

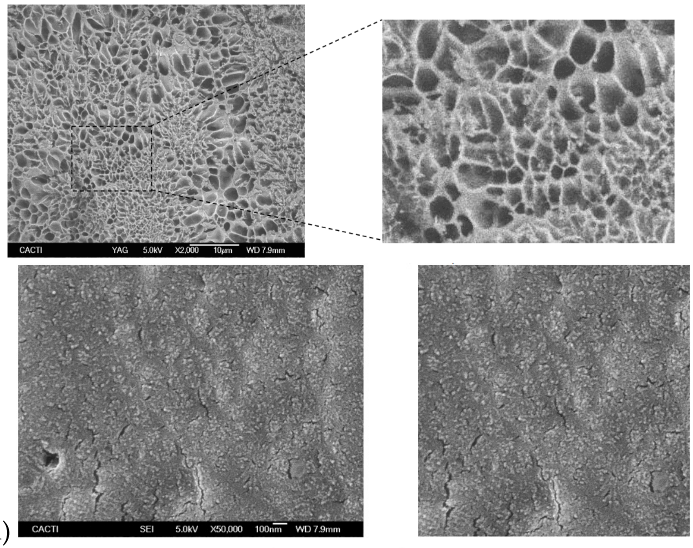


a)

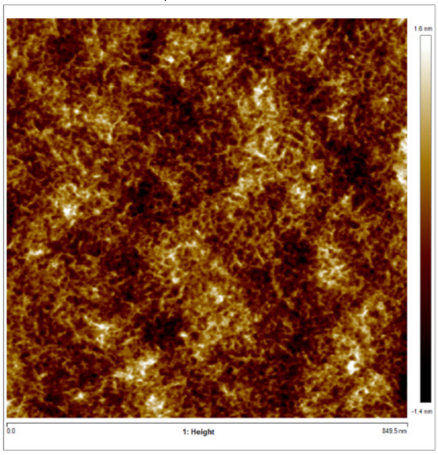


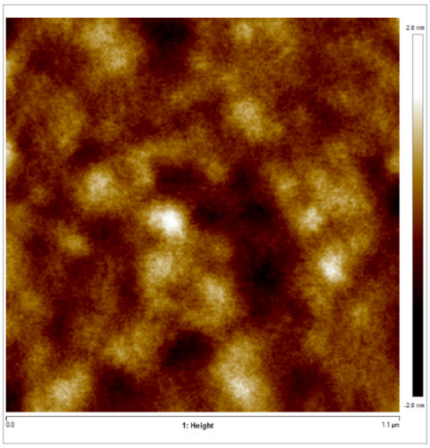


(b) (c)

**Figure S12.** (**a**) Cryo-SEM images; (**b)** Image of Zone 1 at a magnification of 0.84X0.84 μm$^2$; (**c**) Image of Zone 2 at a magnification of 1.1X1.1 μm$^2$ of hydrogel gelatin.

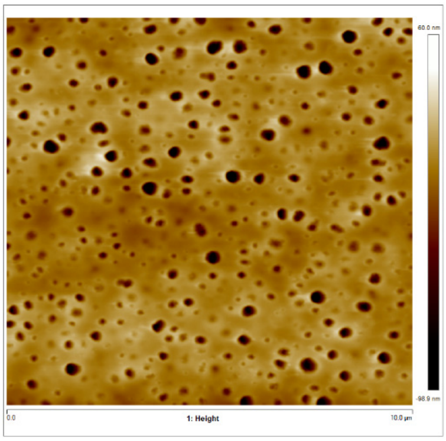


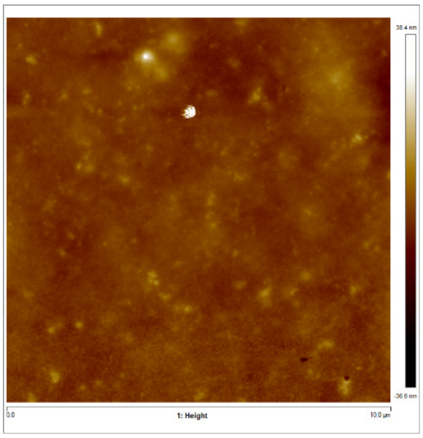


(a) (b)

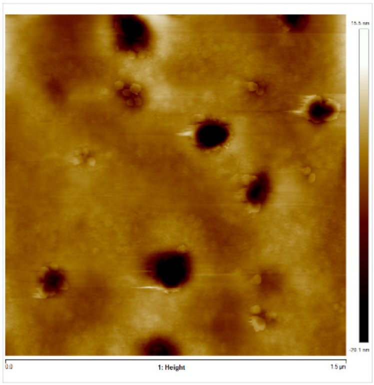


(c)

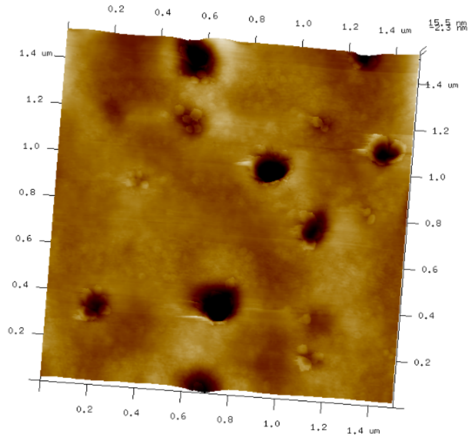

(d)

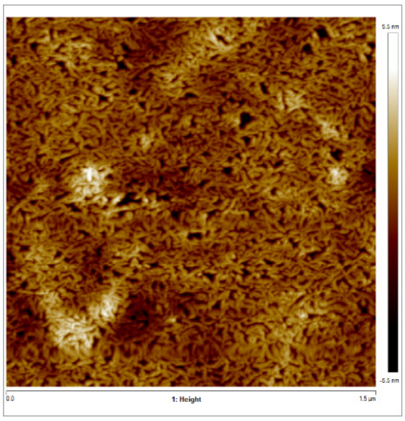


(e)

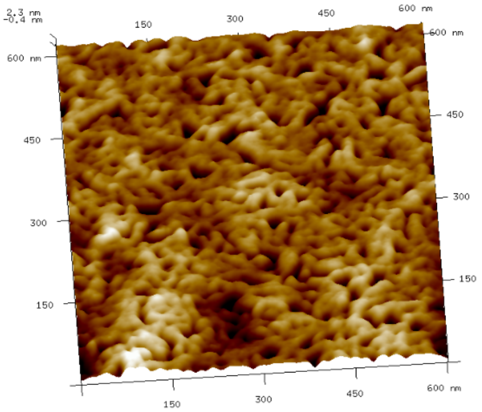

(f)

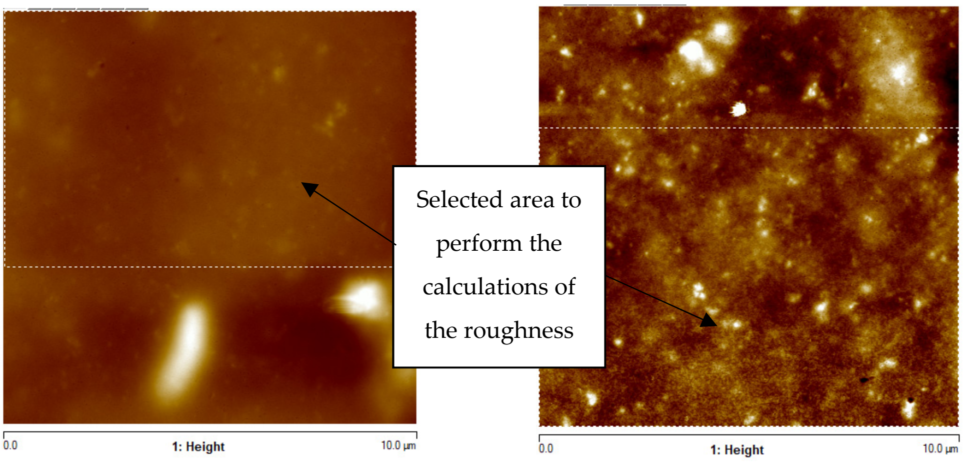


(g) (h)

**Figure S13.** (**a**) Zone 2 of GE/25IL; (**b**) Zone 2 of GE/5IL Topographic 2D images Zone 2, 10x10 μm$^2$; (**c**) Zone 1, Image 2D Field of View 1.5X1.5 μm$^2$ of GE/25IL; (**d**) Zone 1, Image 3D Field of View 1.5X1.5 μm$^2$ of GE/25IL; (**e**) Zone 1, Image 2D, Field of View 1.5X1.5 μm$^2$ of GE/5IL; (**f**) Zone 1, Image 3D Field of View 1.5X1.5 μm$^2$ of GE/25IL; (**g**) GE/5IL of Zone 1; (**h**) GE/5IL of Zone 2.

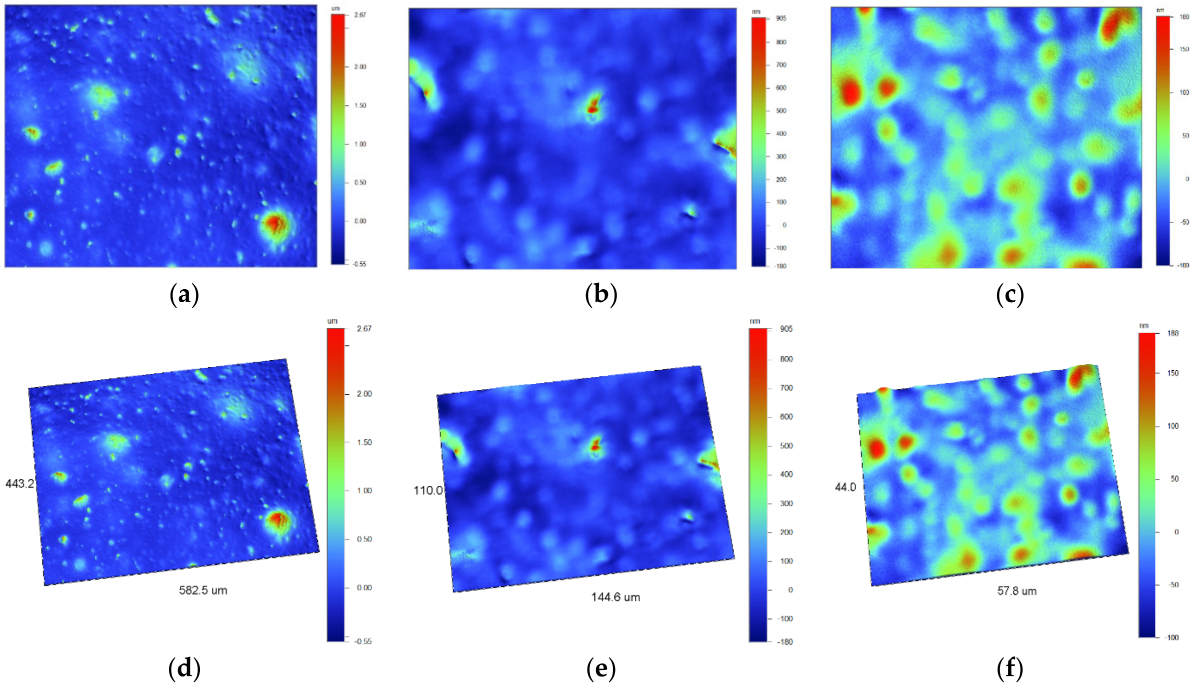

**Figure S14.** Imaging of WLOP method. (**a**) 2D image at 5x_2x (443.2X582.5 μm²; (**b**) 20x_2x (110X144 μm²; (**c**) 250x_2x (44X58 μm² ); (**d**) 3D magnification 5X_FOV 2X (443.2X582.5 μm²; (**e**) 3D magnification 20X_2X (110X144μm²), (**f**) 3D magnification 50X_2X (44X58μm²).

The development of the calculations of the three-dimensional roughness parameters (Sa, Sq, Sz, Ssk and Sku), have been developed in previous studies [1].

**Table S8.** Roughness parameter values obtained by WLOP of shark gelatin hydrogel.

| **INTERFEROMETRIC OPTICAL PROFILOMETRY / 3D Amplitude Roughness Parameters** | | | | | |
|---|---|---|---|---|---|
| | **Sa (nm)** | **Sq (nm)** | **Sz (nm)** | **Ssk** | **Sku** |
| **5X_2X** | 148.07 | 241.84 | 2160.00 | 3.53 | 22.76 |
| **20X_2X** | 54.31 | 80.55 | 780.03 | 2.53 | 16.66 |
| **50X_2X** | 27.73 | 37.31 | 251.27 | 1.14 | 5.12 |

The gelation point of the Ionogles was determined by monitoring the loss and storage moduli, as well as the complex viscosity (Figure S15). A transition in the complex viscosity occurs in the thermal range between 15 °C and 22 °C as well as in the storage and loss moduli during the heating ramp, which is indicative of the interruption of the elastic network of the hydrogel and of in agreement with the sol/gel transition observed for the gelatin hydrogel and which is slightly lower for the Ionogels.

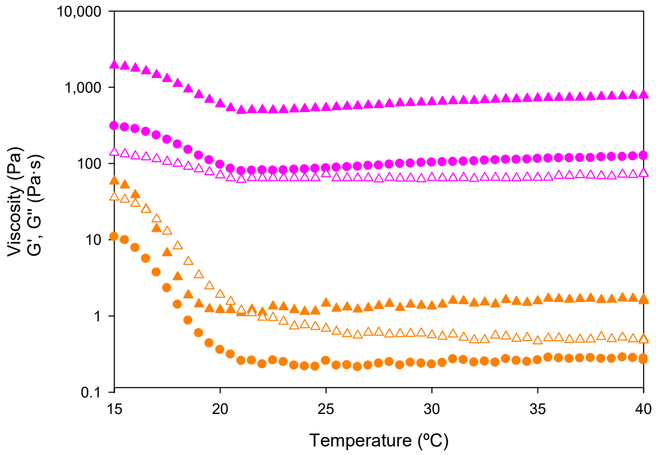


**Figure S15.** Complex viscosity variation (filled ○); and storage modulus (G', filled Δ) and loss modulus (G'', open Δ) versus temperature of gelatin hydrogel (orange color) and Ionogel (fuchsia color).
